# Relating psychiatric symptoms and self-regulation during the COVID-19 crisis

**DOI:** 10.1038/s41398-022-02030-9

**Published:** 2022-07-11

**Authors:** Matilde M. Vaghi, McKenzie P. Hagen, Henry M. Jones, Jeanette A. Mumford, Patrick G. Bissett, Russell A. Poldrack

**Affiliations:** 1 Department of Psychology, Stanford University, Stanford, CA USA; 2 Department of Psychology, University of Washington, Seattle, WA USA; 3 Department of Psychology, University of Chicago, Chicago, IL USA

**Keywords:** Human behaviour, Diagnostic markers, Depression, Psychiatric disorders

## Abstract

Disruptions of self-regulation are a hallmark of numerous psychiatric disorders. Here, we examine the relationship between transdiagnostic dimensions of psychopathology and changes in self-regulation in the early phase of the COVID-19 pandemic. We used a data-driven approach on a large number of cognitive tasks and self-reported surveys in training datasets. Then, we derived measures of self-regulation and psychiatric functioning in an independent population sample (*N* = 102) tested both before and after the onset of the COVID-19 pandemic, when the restrictions in place represented a threat to mental health and forced people to flexibly adjust to modifications of daily routines. We found independent relationships between transdiagnostic dimensions of psychopathology and longitudinal alterations in specific domains of self-regulation defined using a diffusion decision model. Compared to the period preceding the onset of the pandemic, a symptom dimension related to anxiety and depression was characterized by a more cautious behavior, indexed by the need to accumulate more evidence before making a decision. Instead, social withdrawal related to faster non-decision processes. Self-reported measures of self-regulation predicted variance in psychiatric symptoms both concurrently and prospectively, revealing the psychological dimensions relevant for separate transdiagnostic dimensions of psychiatry, but tasks did not. Taken together, our results are suggestive of potential cognitive vulnerabilities in the domain of self-regulation in people with underlying psychiatric difficulties in face of real-life stressors. More generally, they also suggest that the study of cognition needs to take into account the dynamic nature of real-world events as well as within-subject variability over time.

## Introduction

The psychological construct of self-regulation broadly refers to a range of abilities that enable flexible and goal-directed behavior. Together with related concepts such as cognitive control and impulsivity, it has been associated to numerous real-world outcomes such as academic performance, health, and economic well-being [1]. Distortions of self-regulation are a hallmark of numerous psychiatric disorders, including schizophrenia, depression, and obsessive-compulsive disorders [2]. Substantial evidence demonstrates disruption of cognitive constructs relevant for self-regulation [3–7] as well as the associated neural circuitry [8, 9] across several psychiatric conditions. Additionally, environmental demands such as physiological and psychological stress are thought to impair cognitive functions implicated in self-regulation [10, 11]. Even though it remains unclear to what degree laboratory manipulations can generalize to real-world stress, a large set of studies have identified the ways in which stress affects self-regulation [11–16].

The rapidly evolving situation associated with the outbreak of the SARS-CoV-2 virus causing the COVID-19 pandemic was characterized by extreme uncertainty and fear of potential infection, likely to increase perceived stress and anxiety. Regardless of the nature of the disaster, traumatic, natural, or environmental crises aggravate depression, posttraumatic stress disorders, as well as substance abuse [17]. Additionally, in the case of the COVID-19 pandemic, containment measures implemented to reduce the spread of the virus mainly included social distance and self-isolation, which are known as risk factors for mental health issues [18]. For example, recent work has shown transient volumetric brain change patterns in regions commonly associated with stress and anxiety occurring following the initial outbreak of the COVID-19 pandemic. Those changes were associated with the amount of time elapsed from lockdown relief [19]. Critically, in the early phases of the pandemic, individuals had to adapt quickly to a novel situation and employ a degree of cognitive and behavioral flexibility to adjust to modifications in daily routines and circumstances, due to changes in national behavioral patterns as well as shutdowns of usual day-to-day functioning. Hence, the pandemic provided an unprecedented opportunity to track the relationship between an unfolding crisis and self-regulation, in an ecological fashion, rather than relying on artificial manipulations generally used in laboratory experiments [20].

While extensive research has documented the effect of the pandemic on mental health [21–26], limited information is currently available on its impact on cognitive mechanisms supposedly relevant for flexible adaptation. Our study set out to examine the role of individual differences in psychiatric symptoms in relation to changes in self-regulation during the early stages of the COVID-19 pandemic. Understanding such consequences can shed light on cognitive mechanisms vulnerable to real-life stressors, especially for people with underlying psychiatric difficulties.

To address this question, we took advantage of an existing cohort that had been previously examined on a broad battery of self-regulation entailing 37 computer-based cognitive tasks as well as 22 self-reported surveys [27], providing a baseline for self-regulation before the onset of the pandemic. A subset of these individuals was invited to complete the entire battery again during the unfolding of the initial phase of the pandemic (5th May 2020–11th June 2020), at which point they also provided additional information regarding psychiatric symptomatology. Additionally, on this second occasion, they also reported on their wellbeing and the subjective impact of the COVID-19 pandemic.

Adoption of this large battery overcomes some of the limitations of traditional approaches, which generally rely on selecting a specific task (or a small subset) and do not account for the possible heterogeneity between patients within a given diagnostic entity. They also leave untested the hypothesis that deficits observed across different tasks might be due to the same underlying dysfunctional mechanism. Here, we used an extensive multidimensional battery of tasks and self-report surveys that aimed at dissecting and quantifying several constructs associated with self-regulation, rather than focusing on a specific one. In order to identify comprehensive cognitive factors, each capturing specific processes of relevance for successful self-regulation, we used Exploratory Factor Analysis (EFA) on training datasets to derive latent orthogonal dimensions of self-regulation. These have been shown to possess stability over time [28], overcoming some of the challenges of obtaining robust individual differences from cognitive paradigms [28–30]. Similarly, an analysis was conducted on psychiatric symptom questionnaires to uncover transdiagnostic dimensions of psychiatric symptoms [31, 32].

Using scores derived from psychiatric symptoms examined transdiagnostically, we show that core dimensions of psychopathology were related to a differential cognitive response in the face of the emerging COVID-19 pandemic. We show that in spite of a statistical relationship between psychiatric symptoms and cognitive tasks, the latter fail to predict substantial variance in psychiatric symptoms, challenging the possibility of using cognitive tasks to predict mental health outcomes. In contrast, the predictive success of self-reported measures of self-regulation revealed the psychological dimensions relevant for separate transdiagnostic dimensions of psychiatry. Overall, these results show that people with high psychiatric traits were characterized by changes in self-regulation cognitive functions during the emergence of the COVID-19 pandemic.

## Materials and methods

### Participants

Subjects were recruited through Amazon Mechanical Turk participant tool (MTurk). Testing was administered using the Experiment Factory Platform [33], which enables collection of behavioral measures on MTurk in multiple sessions, as necessitated by our long behavioral battery. Data from 102 participants passed quality check criteria which were defined by [27] and extended to further screen our participants (Supplementary Material). Hence, our longitudinal analyses were based on data collected on 102 subjects who provided data on self-regulation pre (i.e., 2016) and post (i.e., 2020) the onset of the COVID-19 pandemic. See Supplementary Material for further details on data collection and training datasets. Table S1 reports demographic data of subjects included in the training and testing datasets. The study was approved by the Stanford Institutional Review Board (Protocol number: 55844). Participants read an informed consent and agreed on participation.

### Measures collected

Our battery for self-regulation mirrored the one used by [27] and included 37 behavioral tasks and 22 self-report surveys. Tables S11 and S12 give an overview of tasks and surveys of self-regulation as well as the corresponding derived variables. Derived variables of self-regulation reflected measures such as temporal discounting and impulsivity as well as more generic cognitive domains such as working memory, cognitive flexibility, information processing as further described in the Supplementary Material. A full description of self-regulation measures is reported in [27]. Procedures for the selection of self-regulation variables and data cleaning for both the training and testing datasets are specified in the Supplementary Materials.

In order to investigate a range of psychiatric symptoms we asked participants to complete a host of self-report questionnaires. These included: Self-Rating Depression Scale (SDS) [34], Short Scales for Measuring Schizotypy (SSS) [35], Obsessive Compulsive Inventory Revised (OCI-R) [36], Leibowitz Social Anxiety Scale (LSAS) [37], State Trait Anxiety Inventory (STAI) [38], Apathy Evaluation Scale (AES) [39], Eating Attitude Test (EAT-26) [40], Barratt Impulsiveness Scale (BIS-11) [41], and Alcohol Use Disorder Identification Test (AUDIT) [42]. The selection of these surveys was based on previous studies which used these measures to derive parsimonious latent transdiagnostic psychiatric factors [31, 32].

Additionally, to estimate changes in wellbeing related to the emerging pandemic we asked participants to complete the Short Scale for Measuring Loneliness [43], the Perceived Stress Scale [44], and the Multidimensional Scale of Perceived Social Support [45]. These measures were rated twice, with reference to a period before and after the outbreak of the SARS-CoV-2 virus. We also used the Corona Health and Impact Survey (CRISIS) [46] which measured “Covid worries” (e.g., how worried have been during the past 2 weeks about infection), “life changes” (e.g., subjective impacts of structural changes such as changes in social contacts), “mood states” (including ten items from the circumplex model of affect), and “daily behaviors” (e.g., frequency of exercise, sleep duration or media use) in relation to the COVID-19 outbreak. Finally, we measured mindset towards the global/societal impact caused by the outbreak of COVID-19 (Zion et al., in prep) and mindset towards stress [47] to investigate those as moderators when evaluating the subjective impact of COVID-19.

### Study design and procedure

**Fig. 1:**
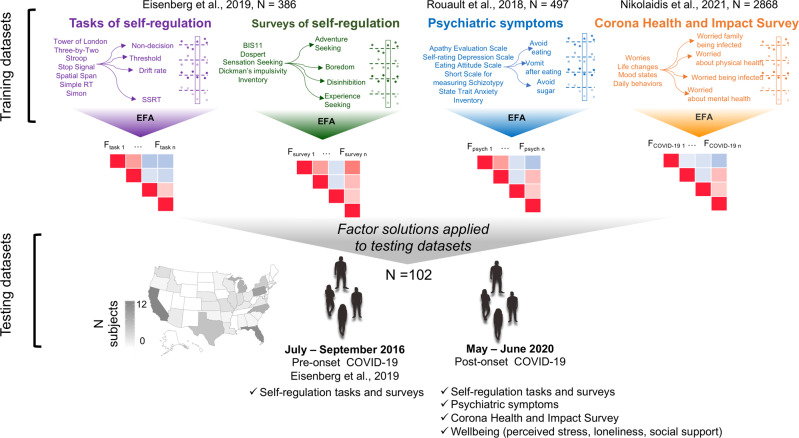
Study design and experimental procedure. We used data from published studies as training datasets. The training datasets included measures of self-regulation [27], psychiatric symptoms [32], and the impact of the COVID-19 pandemic [46] which were investigated in the current study. We used Exploratory Factor Analysis (EFA) for each set of variables, and we applied the obtained factor solutions to our independent testing datasets. Testing datasets included 102 participants who completed tasks and surveys of self-regulation twice; before (July–September 2016; [27]) and after (May–June 2020) the onset of the COVID-19 pandemic. Data collection after the onset of COVID-19 additionally included a large battery of questionnaires investigating psychiatric symptoms as well as the impact of COVID-19, which was assessed via the Corona Health and Impact Survey [46]. Finally, measures of wellbeing (i.e., perceived stress, loneliness, social support) were collected after the onset of COVID-19. However, no training dataset was available for this set of measure. Hence, a different analysis pipeline was used (see “Material and methods”). Amazon Mechanical Turk was used for data collection. The location of participants in our testing datasets is illustrated on the map.

We had a two-step analytical approach. Briefly, we used data from previously published studies as training datasets to generate factor structures (Fig. 1). On all the training datasets, we used maximum likelihood estimation to perform EFA, followed by *oblimin* rotation to rotate the factors without enforcing orthogonality. Factor scores were estimated using the *tenBerge* method [48]. In line with previous investigations, EFA was applied to the self-regulation variables [27] and at the item-level for psychiatric measures [31, 32] and the CRISIS questionnaire [46]. All analyses were implemented using the *fa* function from the psych package in R [49]. In order to determine the number of factors to extract, we relied on methods used for each set of variables by previous investigators. Namely, for the self-regulation measures and in the case of the CRISIS questionnaire, we used the Bayesian Information Criteria (BIC) which selects the number of factors to extract taking into account both model complexity and the ability to capture the data. We extracted 3 factors for psychiatric symptom measures, as a previous study on the training dataset used here found that, according to the Cattell’s criterion, a model with three underlying factors provided the best account of the covariance [32]. Finally, we used *predict* from the psych package in R to estimate factor scores on our testing datasets, based on the EFA solution on the training ones. Factor solutions obtained on the training datasets were used to predict the factor scores of the independent pool of subjects (*N* = 102) tested twice, in a longitudinal fashion, before (i.e., 2016) and after (i.e., 2020) the onset of the pandemic. Thus, the factor analysis solutions used to estimate factor scores for longitudinal analysis were derived from an independent sample, avoiding any potential circularity.

We employed linear mixed models to examine longitudinal changes of self-regulation, using the *lme4* [50] and *lmerTest* [51] packages in R. Factor scores estimated on our testing datasets from the EFA solution (based on independent training data) represented our dependent variables. In all these models, time was coded as −0.5 (pre-covid)/0.5 (post-covid), so that the main effects for between-subjects covariates reflect the average time effect. Further, between-subject variables were mean centered and scaled by the standard deviation. Therefore, parameter estimates for these variables reflect changes in standard deviation units, and the main effect of time and intercept refers to the average value of the between-subject variables. All statistical tests were two-sided. For longitudinal and cross-sectional analyses, significance values were FDR corrected over the number of dependent variables tested within each set of models. Full details are provided in the Supplementary Materials.

## Results

### Exploratory factor analysis on training datasets

To identify a latent structure pointing to dissociable factors, we used EFA on each of our training datasets (Fig. 1 and Supplementary Materials). Even though the method used here is one of the many possible methods that could have been used for dimensionality reduction, we define this approach as ‘data-driven’ because the dimensionality reduction is based on the structure of the data themselves rather than on prior psychological theory. In the case of self-regulation, 61 variables from surveys or 113 variables from tasks reflected means of specific item sets, comparisons between task conditions, or model parameters thought to capture psychological constructs (Fig. 1). Similarly, EFA was applied to each of the training datasets pertaining to psychiatric symptoms and to the Corona Health and Impact Survey (CRISIS). For each EFA, overall model fit was satisfactory (RMSEA < 0.08) [52] (Table S2), and the moderate correlations among the factor scores (Pearson’s correlation < 0.5 [53]) within each training set suggested that they reflected largely independent constructs (Fig. S5).

**Fig. 2:**
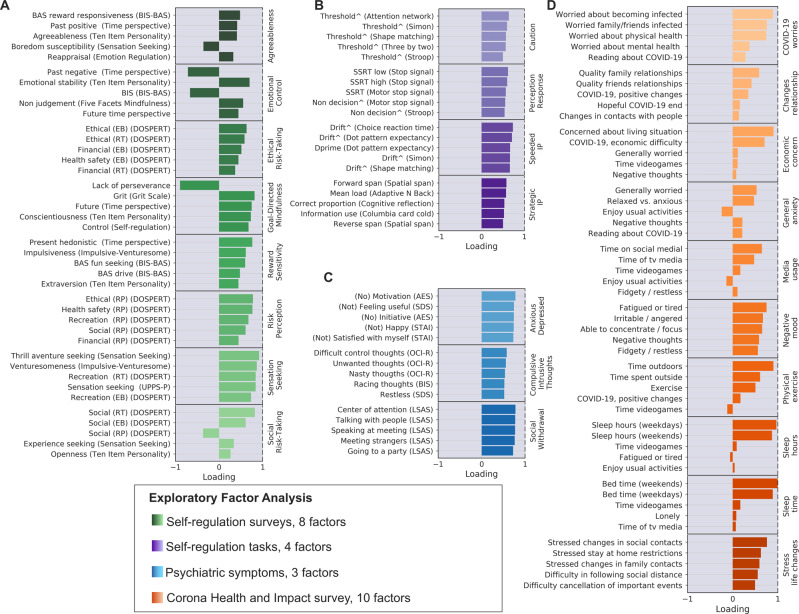
Exploratory Factor Analysis. Summary of Exploratory Factor Analysis (EFA) conducted on the training datasets for self-regulation surveys (**A**), self-regulation tasks (**B**), psychiatric symptoms (**C**), Corona Health and Impact survey (**D**). For each factor, the five items loadings more strongly are shown. The complete matrix of loadings is displayed in the online Jupyter Notebook. The height of the bar reflects the loading. Color codes indicate the subset of variables on which EFA was performed. ^ indicates a parameter from the Diffusion Decision Modeling. BAS Behavioral Activation System subscale; BIS Behavioral Inhibition System subscale; EB expected benefits subscale; RT risk taking subscale; RP risk perception subscale; AES Apathy Evaluation Scale; SDS Self-rating Depression Scale; STAI State Trait Anxiety Inventory; OCI-R Obsessive Compulsive Inventory Revised; LSAS Leibowitz Social Anxiety Scale.

A parsimonious latent structure of 4 and 8 factors was identified for tasks and surveys of self-regulation, respectively, based on the Bayesian Information Criterion. Similarly, a 10 factor solution was obtained from the CRISIS survey (Fig. S2). Finally, 3 psychiatric dimensions were obtained from the set of questionnaires investigating psychiatric symptoms. Interpretation of the factor solutions was based on the strongest individual loadings (Fig. 2).

Factors for the survey model reflected a combination of closely related variables, deriving from different surveys aimed at measuring overlapping constructs. For example, variables that strongly loaded on the Sensation Seeking factor derived from the Sensation Seeking Scale, the UPPS-P Impulsive Behavior Scale, and the Domain Specific Risk taking scale. In contrast, an heterogenous set of variables determined the nature of a few factors such as in the case of the Emotional Control factor incorporating measures related to emotional stability, future time perspective, eating behavior, and behavioral inhibition. Similarly, the Goal-Directed/Mindfulness factor relates to perseverance, grit, conscientiousness, self-control, and mindfulness (Fig. 2A). For EFA on self-regulation tasks, Strategic Information Processing (IP) captured high order strategies as variables loading on this factor related to working memory, risk taking, and model-based decision making. In contrast, Speeded IP, Caution, and Perception/Response factors related to speeded decision-making tasks and captured separate parameters estimated using the diffusion decision model (DDM), namely drift rate, threshold, and non-decision time, respectively (Fig. 2B). Therefore, the identified factors recapitulate computational parameters which are likely correlated across tasks, but tap onto different cognitive processes. The use of factor scores alleviates the challenge of obtaining robust measurements from the individual cognitive paradigms.

In agreement with previous work [31, 32], the EFA solution for psychiatric questionnaires led to the identification of factors reflecting Anxious-Depression (AD), Compulsive behavior, and Intrusive Thoughts (CIT), and Social Withdrawal (SW) phenotype based on the strongest individual item loadings (Fig. 2C).

The EFA solution on the CRISIS questionnaire isolated 10 factors generally indexing mood (i.e., COVID-19 Worries, General Anxiety, Negative Mood), life-changes (i.e., Changes relationship, Economic concern, Stress life changes), and daily behavior (i.e., Media usage, Physical exercise, Sleep hours, Sleep time) related to the period following the onset of the pandemic (Fig. 2D).

### Effect of the pandemic outbreak on task-based self-regulation measures

We first tested the hypothesis that individual differences in psychiatric symptoms related to changes of self-regulation in response to the emergence of COVID-19. To this aim, we used factor scores of self-regulation as dependent variables in the context of linear mixed models, which included transdiagnostic psychiatric dimensions as regressors of interest, while systematically controlling for the effect of age, gender, and IQ (Table S3 and Supplementary Materials).

**Fig. 3:**
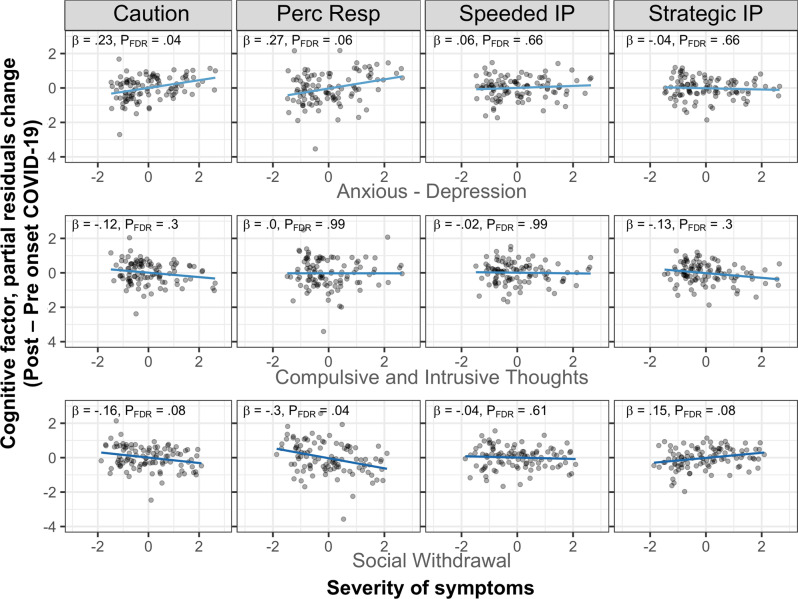
Relationship between psychiatric dimensions and within-subject self-regulation changes. The plots represent within-subject change in cognitive factor from before to after the onset of the pandemic (*y*-axis) in relation to the severity of different symptoms dimensions (*x*-axis). The first, second, third, and fourth column represents within-subject change for the Caution, Perception Response, Speeded IP, Strategic IP factor, respectively. Severity of Anxious-Depression symptoms related to within-subject change in the Caution factor from before to after the onset of the pandemic. Higher values on the Caution factor index the need to accumulate more evidence until a response is executed. Severity of Social-Withdrawal symptoms related to a decrease on the Perception/Response factor after the onset of the pandemic, indexing faster perceptual and motor execution processes. As our outcome measures did not contain any missing data for any subjects, a two stage model can be used for visualization purposes [101]. Firstly, we computed the paired difference for the dependent variable of interest. Then, we fit a linear model to the paired difference. Hence, the partial residuals, controlling for the effect of variables in the model, besides each of the predictor (i.e., AD), were plotted (effect_plot from jtools package in R [102] was used for this visualization). The linear relationship between the change score and the psychiatry symptoms displayed here is conceptually identical to the interaction effect of the model described in the main text. All the psychiatric dimensions were entered in the same model, which also controlled for the influence of age, gender, and IQ as explained in the “Materials and methods” section. *P*-values reported on the figure refer to the main analysis described in the main text and reported in Table S3. *P*-values for each effect of interest (e.g., interaction effect, AD × Time _pre/post_ on Caution) are FDR-corrected for multiple comparisons over the number of dependent variables tested (*N* = 4). We ascertained that FDR-corrected *P* values remained significant even after the exclusion of potential influential cases as described in the Methods section. Results were robust to different analytical approaches. All the individual data points are shown in the plots.

Longitudinal changes in self-regulation were related to individual differences in transdiagnostic psychiatry symptoms. We found that the Anxious-Depression psychiatric factor related to longitudinal changes on the Caution self-regulation task factor (β [95% CI] = 0.23 [0.06, 0.40]; *P*_unc_ = 0*.*01*, P*_FDR_ = 0.04) (Fig. 3). In contrast, the Social-Withdrawal psychiatric factor was associated with within-person changes on the Perception/Response self-regulation task factor (β [95% CI] = −0.30 [−0.52, −0.07]; *P*_unc_ = *.01, P*_FDR_ = 0.04). These results remained significant even after the exclusion of influential cases as described in the Supplementary Material (Caution: β [95% CI] _AD*time_ = .21 [0.05, 0.35]; *P*_unc_ = 0.01*, P*_FDR_ = 0.02; Perception/Response: β [95% CI] _SW*time_ = −0.31 [−0.50, −0.12]; *P*_unc_ < 0.001*, P*_FDR_ < 0.001). We also used an analysis of covariance conditioning on baseline [54] to confirm that our results were not due to the specific analytical approach adopted. In this set of analyses, we used the self-regulation factor score following the onset of COVID-19 as dependent variable with the baseline measurement (i.e., self-regulation before the onset of COVID-19) as a covariate together with age, gender, IQ, and each of the psychiatric factors. Results confirmed that the Anxious-Depression phenotype related to longitudinal changes on the Caution factor (*β* [95% CI] = 0.26 [0.10, 0.43]; *P*_unc_ < 0.001*, P*_FDR_ < 0.001). Similarly, there was an effect of the Social-Withdrawal psychiatric factor on longitudinal changes on the Perception/Response (β [95% CI] = −0.26 [−0.47, −0.05]; *P*_unc_ = 0.02*, P*_FDR_ = 0.04). Hence, higher scores on the Anxious-Depression phenotype related to a larger increase in within-subject change over time in cautious responding, where a higher threshold (i.e., more cautious responding) was observed during the pandemic (compared to pre-pandemic). There was also indication that a Social-Withdrawal dimension corresponded to faster stimulus encoding and motor processes during the pandemic. Apart from an effect of these two psychiatric factors, all the other regressors did not have a significant effect on changes in self-regulation, as also shown via equivalence testing (Fig. S6).

As expected, there were main effects of IQ and age (Table S3). Higher IQ was associated with better performance on the Strategic IP, and with faster decision time (Speeded IP). Older age was associated with worse performance on the Strategic IP and increased Caution.

### Effect of pandemic outbreak on survey-based self-regulation measures

In a separate set of models, mirroring those used for cognitive factors of self-regulation, we tested whether pre to post pandemic changes in survey-based self-regulation were moderated by individual differences. As expected given that these measures of self-regulation are thought to index largely stable traits, the sudden onset of the pandemic did not cause a change on those measures (Table S4). Equivalence testing (“Material and methods”) confirmed that the effects of the interactions were smaller than the smallest effect of interest (Fig. S7). In contrast, robust main effects of the transdiagnostic psychiatric symptoms were identified.

Impoverished emotional control could be observed across psychiatric dimensions. Accordingly, higher values on the Anxious-Depression (β [95% CI] = −0.43 [−0.56, −0.30]; *P*_unc_ < 0*.*001*, P*_FDR_ < 0.001), Compulsive (β [95% CI] = −0.24 [−0.36, −0.13]; *P*_unc_ < 0*.*001*, P*_FDR_ < 0.001), and Social-Withdrawal (β [95% CI] = −0.41 [−0.53, −0.29]; *P*_unc_ < 0.001*, P*_FDR_ < 0.001*)* psychiatric factor related to lower emotional control. However, selective profiles of self-regulation were also identified for each psychiatric dimension. In particular, the Anxious-Depression factor was significantly associated with lower Goal Directed-Mindfulness (β [95% CI] = −0.76 [−0.93, −0.58; *P*_unc_ < 0.001*, P*_FDR_ < 0.001), Agreeableness (β [95% CI] = −0.50 [−0.71, −0.30]; *P*_unc_ < 0.001*, P*_FDR_ < 0.001), and Risk Perception (β [95% CI] = −0.34 [−0.56, −0.12]; *P*_unc_ < 0.001*, P*_FDR_ < 0.001). In contrast, the Compulsive behavior and Intrusive Thoughts factor was significantly associated with increased Ethical Risk-Taking (β [95% CI] = 0.29 [0.10, 0.47]; *P*_unc_ < 0*.*001*, P*_FDR_ < 0.001). Sensation Seeking, Reward Sensitivity and Social Risk-Taking exhibited the reverse relationship with symptom clusters: they were increased in subjects with higher scores on the Compulsive behavior and Intrusive Thoughts factor and decreased in subjects with higher scores on the Social-Withdrawal factor (all *P*_FDR_ < 0.001). Female gender was related to lower scores on the Emotional Control, Sensation Seeking, and Ethical Risk Taking factors.

### Trajectories of wellbeing related to the pandemic onset

To quantify the dynamics of wellbeing as COVID-19 reverberated across the U.S., we asked participants to self-report their current stress, loneliness, and perceived social support during the emerging phase of the pandemic (5th May 2020–11th June 2020). We also asked the same questions in relation to the period preceding the onset of COVID-19; note that this assumes that memory for previous mental states is unaffected by current mental states, which is often not the case [55]. We modeled longitudinal changes in stress, loneliness, and perceived social support as a function of demographic characteristics, psychiatric symptoms, and mindset. The non-significant interactions between our variables and time (all *P*_FDR_ > 0.09, Table S5 and Supplementary Material) suggested that none of them were associated with longitudinal changes on perceived stress, loneliness, and social support. Equivalence testing confirmed that the effects of the interactions were smaller than the smallest effect of interest (Fig. S8).

**Fig. 4:**
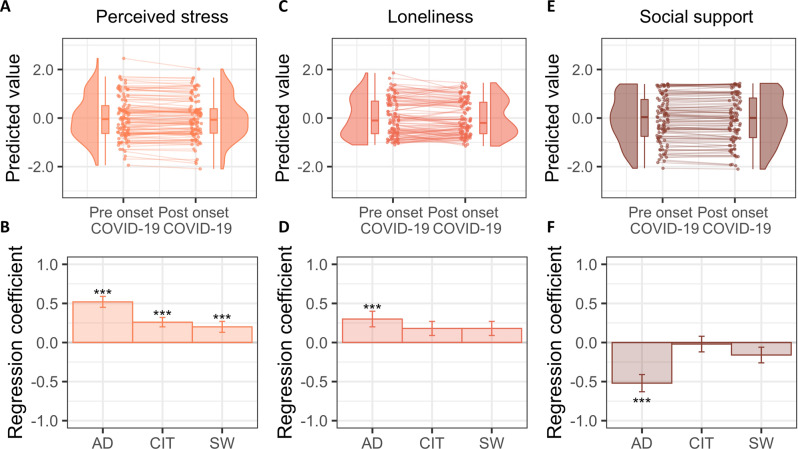
Trajectories of wellbeing in relation to the pandemic onset. A series of longitudinal models was conducted to examine psychological and psychosocial changes in correspondence with the onset of the pandemic. In the top row, predicted values from the respective longitudinal model are shown for each of the dependent variables. Predicted, rather than raw values are shown, to account for the covariates included in the models. The predict function in R was used to obtain the predicted value based on the linear model implemented. Perceived stress (**A**), loneliness (**C**), and social support (**E**) did not vary as a function of the pandemic onset. In the bottom row, association between perceived stress (**B**), loneliness (**D**), and social support (**F**) and each of the psychiatric dimensions is displayed. Higher levels of psychopathology were related to increased perceived stress. Additionally, an AD psychiatric symptom dimension related to increased loneliness and reduced perceived social support. For each dependent variable, all the psychiatric dimensions were entered in the same model, which also controlled for the influence of age, gender, IQ as well as mindset attitudes as explained in the Methods section. Hence, the regression coefficients reflect adjusted values. The *y*-axis indicates the change in the dependent variable for each change of 1 SD of symptom scores. Error bars denote SE. **P_FDR_ < 0.01, ***P_FDR_ < 0.001. *P*-values for each effect of interest (e.g., main effect of AD phenotype on perceived stressed) are FDR-corrected for multiple comparisons over the number of dependent variables tested (*N* = 3). AD Anxious-Depression; CIT Compulsive behavior and intrusive thoughts; SW Social Withdrawal. See also Table S5.

Our results also indicated that there was no effect of the pandemic’s onset on wellbeing. In fact, there was no main effect of time on perceived stress, loneliness, nor social support in the period following the onset of COVID-19, compared the period preceding the pandemic (all *P*_FDR_ > 0.9, Fig. 4A, C, E). This may reflect stability in those traits or biased memory retrieval for the previous timepoint [55, 56].

These models, which included all three psychiatric factors scores and controlled for age, gender, and mindset attitudes, showed that higher levels of Anxious-Depression, Compulsive behavior and Intrusive Thoughts, and Social-Withdrawal corresponded to increased perceived stress (averaged across both time points) (Fig. 4B and Table S5). Additionally, an Anxious-Depression symptom dimension was associated with higher loneliness and diminished perceived social support (Fig. 4D, F and Table S5).

### Subjective impact of the pandemic onset

Finally, we aimed at evaluating the subjective impact of the pandemic onset in the context of a cross-sectional analysis using factors derived from the CRISIS questionnaire [46]. Namely, each factor score from the CRISIS questionnaire was the dependent variable of a general linear model which included several covariates of interest (i.e., demographic characteristics, psychiatric symptoms, and mindset) (Table S6). Overall, higher scores on the psychiatric dimensions were associated with several factors from the CRISIS questionnaires, suggesting a negative impact of the pandemic for people with higher psychiatric traits.

More specifically, an Anxious-Depression phenotype was characterized by a worsening of relationships with family and friends (Table S6). Subjects with high compulsivity traits experienced high COVID-19-related worries (e.g., worries of becoming infected) as well as high stress related to life changes induced by the pandemic (Table S6). High Compulsivity was also linked to high economic difficulty, high media usage, and high physical exercise during the pandemic period (Table S6). Both Social-Withdrawal and Compulsivity related to higher values on the general anxiety factor (Table S6). High negative mood states were found in association with an Anxious-Depression, Compulsive behavior and Intrusive Thoughts, and the Social Withdrawal psychiatric factor (Table S6).

Mindset attitude was also associated with the subjective impact of the pandemic. We found that a catastrophic mindset attitude towards the pandemic was associated with increased COVID-19 worries as well as reduced sleep hours per night (Table S6). A more positive mindset towards stress related to an improvement of relationships with family and friends during the emergence of the pandemic (Table S6).

### Prediction of psychiatric symptoms

We next sought to assess whether measures of self-regulation can successfully predict psychiatric symptoms during the COVID-19 pandemic (Supplementary Material). To conduct a preliminary evaluation in this direction, we used factors of self-regulation derived from tasks or surveys to predict transdiagnostic psychiatric dimensions. Our primary analysis used transdiagnostic measures of psychopathology which in other contexts have demonstrated superior value compared to the individual variables from questionnaires of psychopathology [31, 32]. Secondary analyses using the latter did not change the overall interpretation (Tables S7–S10; see also Fig. S13 displaying the contribution of each survey factor score to individual variables from questionnaires of psychopathology).

As psychiatric symptoms were assessed after the onset of the pandemic but self-regulation was measured before and after, we could test both the prospective and cross-sectional predictive value of self-regulation factors for transdiagnostic dimensions of psychiatry. Namely, to test the prospective predictive value of self-regulation for psychiatric symptoms, we used factor scores computed from data collected before the onset of the pandemic as predictive features. In contrast, factor scores of self-regulation derived from data collected after the onset of the pandemic were used for cross-sectional predictions.

For each analysis, we created two separate predictive feature matrices including either the 8 survey factor scores or the 4 task factor scores. In sample as well as out-of-sample predictions were assessed to predict psychiatric dimensions of interest. We used L2-regularized linear regression using scikit learn, with an internal crossvalidation loop to select the best hyper-parameter. Predictive performance was quantified using *R*^2^ (computed using the sum of squares formulation) and mean absolute error (MAE); note that negative *R*^2^ values in this formulation are reflective of out-of-sample predictions that are less accurate than the sample mean.

Cognitive factors had no predictive ability either prospectively (average cross-validated, *R*^2^ = −0.07, min: = −0.07, max = −0.07; MAE = 0.8, min = 0.73, max = 0.84) or cross-sectionally (average cross-validated, *R*^2^ = −0.06, min: −0.07, max: −0.04; MAE = 0.79, min = 0.71, max = 0.83). Insample predictive analyses failed to reveal significant association as well (Fig. S9). Results remained qualitatively unchanged when using the individual cognitive variables to predict psychiatric symptoms.

**Fig. 5:**
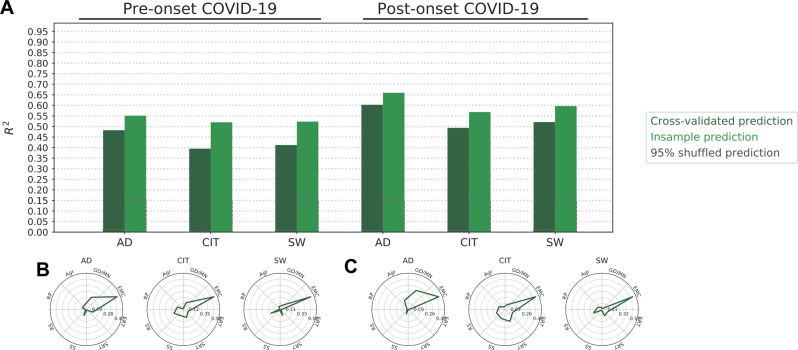
Prediction of psychiatric symptoms dimension using survey factor-scores. **A** Predictions where self-regulation survey-factors scores of the period preceding (pre, prospective) or following (post, cross-sectional) the onset of COVID-19 were used to predict psychiatric symptoms assessed during the initial phase of the COVID-19 pandemic. Fingerprints using factor scores of the period preceding (**B**) or following (**C**) the onset of the COVID-10 pandemic are displayed. Dark and light bars indicate *R*^2^ cross-validated and insample prediction respectively. Dashed gray boxes indicate 95% of null distribution, estimated from 2500 shuffles of the target outcome. Fingerprints displayed as polar plots indicate the standardized β for each factor. The *y*-axes are scaled for each fingerprint to highlight the distribution of associations—no inference can be drawn comparing individual factor magnitudes across outcomes. EMC Emotional Control, GD/MND Goal-Directed/Mindfulness, Agr Agreeableness, RP Risk Perception, RS Reward Sensitivity, SS Sensation Seeking, SRT Social-Risk Taking, ERT Ethical Risk-Taking, AD Anxious-Depression, CIT Compulsive behavior, and Intrusive Thoughts, SW Social Withdrawal.

In contrast to tasks, survey responses were significantly predictive of all transdiagnostic psychiatric dimensions either when considering their prospective (average cross-validated, *R*^2^ = 0.43, min: .39, max: 0.48; MAE = 0.55, min = 0.52, max = 0.58) as well as their cross-sectional (average cross-validated, *R*^2^ = 0.54, min: 0.49, max: 0.6; MAE = 0.45, min = 0.43, max = 0.49) predictive power (randomization test: *P* = 1/2500) (Fig. 5A). We visualized the standardized β coefficients of the predictive models to create a fingerprint representing the contribution of various self-regulation constructs to the final predictive model for a particular psychiatric dimension. Low correlation between the features included in the model allowed interpretation of the resulting fingerprints (Fig. 5B, C). It is evident that Emotional Control (referring to measures such as emotional stability, behavioral inhibition, and emotional eating) is a relevant dimension for all the psychiatric dimensions. In a reduced regression model, Emotional Control was on its own capable of achieving significant in-sample and out-of-sample predictive accuracy, confirming its prominent role compared to other prediction variables (Fig. S12). However, the fingerprints point to the contribution of different self-regulation constructs for specific psychiatric dimensions. For instance, while the AD dimension related to a combination of Agreeableness and Goal-Directed/Mindfulness, the CIT dimension related to Reward Sensitivity, Sensation Seeking, and Social Risk-Taking. The overall fingerprints (i.e., the contribution of each self-regulation construct to each psychiatric dimensions) obtained from prospective (Fig. 5B), and cross-sectional predictions (Fig. 5C) were comparable.

### Prediction of change in health risk behavior

That survey factor scores could accurately predict different psychiatric dimensions aligns with previous work. Eisenberg and colleagues (2019) established that survey-derived factors of self-regulation relate not only to mental health broadly defined, but also to other real-world outcomes such as smoking and drug use. Here, we asked participants to answer questions related to their health risk behavior during the emerging phase of the pandemic (5th May 2020–11th June 2020). As the same questions were asked to the same participants in 2016 we could test the hypothesis that self-regulation factors referring to the period before the onset of the pandemic could predict changes in health risk behavior. We reasoned that these might have been potentially affected by change in routines and lifestyle due to shelter in place restrictions. We implemented a prediction analysis as the one described above. In this case, our dependent variables were represented by derived measures of change in outcome behavior, obtained as described in the Supplementary Methods. Surveys, but not task factor scores, exhibited above-chance prediction for a small number of health risk outcomes (e.g., daily smoking; mental health) (Fig. S11).

## Discussion

Here, we sought to delineate the relationship between dimensions of psychopathology and longitudinal changes in self-regulation in a period that required large-scale behavioral changes.

We show that in the initial phase of the COVID-19 pandemic, changes in self-regulation could be observed in relation to trait-like psychiatric symptoms. We administered a large number of cognitive tasks and personality surveys and adopted a data-driven approach to derive orthogonal dimensions of self-regulation and transdiagnostic factors for psychiatric symptoms. To the best of our knowledge, this study represents the most comprehensive assessment of the relationship between psychiatric symptoms and cognitive self-regulation abilities during the emergence of the COVID-19 pandemic to date.

Our quantitative approach revealed that an Anxious-Depression psychiatric dimension interacted with the onset of the pandemic and related to changes in the Caution cognitive factor, which captures the threshold parameter from the DDM of speeded decision-making tasks. Hence, in those with an Anxious-Depression phenotype, behavior changed in the period after the onset of the pandemic compared to a period preceding the onset of the pandemic, more evidence needed to be accumulated before making a decision. During decision making tasks, humans can strategically prioritize accuracy or speed, resulting in high or low decision thresholds [57]. Accordingly, the threshold parameter can capture the well-known speed-accuracy trade-off in speeded tasks [58]. In this context, participants were not explicitly instructed to emphasize speed or accuracy. However, those with increased values on the Anxious-Depression psychiatric dimension tended towards a more cautious response mode during COVID-19, compared to a period preceding the onset of the pandemic. DDM has been previously applied in affective psychopathology research [59–66], with findings of both increased threshold [60] and reduced drift rate [59, 60] in patients with depression. That an AD psychiatric symptom dimension was associated with changes in decision threshold is consistent with some of these results where clinical depression relates to higher decision threshold [60]. A previous study has also identified a similar trend between decision threshold and an AD dimension. In that case, higher AD was also related to increased meta-cognitive efficiency [32]. A possible relationship between decision threshold and meta-cognitive accuracy has been recently highlighted by a study showing that accumulating evidence resulting in faster decisions for a target accuracy, incurs a cost in meta-cognitive accuracy [67]. Even though such results are suggestive of a link between decision parameters and metacognitive accuracy, how their interaction is perturbed in the case of higher symptoms of depression or anxiety needs to be further investigated.

The examination of a range of cognitive tasks allows generalization of our findings. The Caution factor encapsulates threshold parameters from multiple tasks (e.g., Attention Network Task, Simon, Shape matching, Three by two, Stroop) and points to a potentially more cautious mode that can explain behavior seen across different tasks in depression and anxiety. This finding also highlights the utility of computational models (such as the DDM) that allow behavior to be decomposed into more interpretable components.

Additionally, we found that higher Social-Withdrawal was associated with faster stimulus encoding and motor processes in correspondence with the onset of the pandemic. Results accrued in the literature suggest enhanced perceptual processes in social anxiety disorder [68–70]. Our results align with these findings and support the hypothesis of an amplification of early sensory attention and the idea of a general hyper-vigilance in phobic patients [71], even though previous research mostly related to tasks deploying social stimuli. Overall, our results suggest that situational demands and an increasingly stressful situation could have impacted cognitive functioning, depending on psychiatric dimensions, exacerbating a relationship that could not otherwise be identified before the stresses of the COVID-19 pandemic.

That these processes were impacted under a challenging environmental situation is suggestive of a potentially vulnerable cognitive system in susceptible individuals [72]. We found that distinct psychiatric dimensions are characterized by impairments in different self-regulation domains. Hence, rather than sharing impairments in common dimensions of self-regulation, specificity can be identified for different psychiatric features. Critically, only the AD and SW dimensions displayed cognitive susceptibility in a period associated with stressful life circumstances, while for example the CIT dimension was spared. This might also point to different pathogenic mechanisms for different class of symptoms. Several social and environmental factors, such as for example natural disasters or low social support, have been robustly identified as elements of risk for major depressive disorder [73, 74]. In contrast, no compelling evidence exists on the relationship between psycho-social risk factors and, for example, Obsessive-Compulsive Disorder [75].

However, the ability to directly link alterations of cognitive functioning to an increased stressful situation is limited in the current study by a lack of evidence in relation to an increase in psychological and psychosocial distress during the outbreak of the pandemic. Retrospective reports were the only viable solution for a set of measures aimed at assessing trajectories of wellbeing, as they were not acquired in the first wave of data collection. Due to this methodological limitation, our null results might have been driven by biases linked to retrospective reports, which are vulnerable to different distortions. For example, they are influenced by current mood [56] and, especially in the case of negative mood states, tend to be exaggerated in retrospective ratings [55]. Nevertheless, several other studies which used different approaches to investigate the trajectory of psychological distress in the period corresponding to the emergence of the pandemic were unable to detect significant changes in psychological and psychosocial function in correspondence of the time period investigated in this study. In a nationwide sample of American adults, no significant mean changes in loneliness were found between January and April 2020 [76]. Similarly, during the seven weeks of strict lockdown in the UK, longitudinal assessments revealed that loneliness levels remained relatively stable [77]. Google Trends showed that Google searches for loneliness increased in the month leading up to lockdowns in Western Europe but remained high only for the following fortnight, before returning to usual levels [78]. More generally, longitudinal studies indicated that mental health in UK and USA sample has deteriorated [79, 80], but only for a limited period of time. Trajectories over time revealed that although psychological distress rose in the initial stages of the pandemic in the USA (April 2020), they returned to baseline levels within two months [81]. A similar result was also confirmed by an independent study where the proportion of US individuals reporting serious psychological distress in April 2020 did not significantly differ from that of July 2020 [82]. In USA, a considerable increase in mental health-related Google searches was identified in the period immediately preceding the government’s disposition (i.e., shelter in place, week March 16, 2020), but this quickly stabilized in less than 4 days [83]. Given these multiple tiers of evidence, it is possible that by the time of our data collection (May–June 2020) distress already recovered towards baseline after an initial peak of mental discomfort. A possible untested competing hypothesis is that emerging cognitive difficulties were triggered by increased uncertainty, which is often ill-tolerated in anxiety disorders and depression [84].

Beside changes over time dependent on individual differences in psychiatric dimensions, our results highlighted that older age is associated with an increase in response caution, which is in line with canonical findings of slower decisions in elder people, through an increase in response caution and longer non decision time [85]. We also identified an association such that higher estimated IQ and younger age-related to better Strategic IP, as it has been shown for a host of cognitive tasks measuring high order functions captured by this factor [86].

Our prediction analysis showed that self-regulation cognitive constructs lack predictive power for psychiatric symptoms. In contrast, surveys of self-regulation predicted psychiatric symptoms moderately well. Previous work has shown the merits of transdiagnostic psychiatric scores which capture information over and above the individual constituent scales [31, 32]. However, in this case, prediction power of cognitive measures was not ameliorated by a transdiagnostic approach and predictive performance of tasks was not significantly different when using transdiagnostic measures for psychiatric symptoms or the traditional individual scores.

Even though cognitive tasks have shown successful predictive performance in the case of political attitudes [87], lack of predictive power from cognitive tasks is not surprising. Our results mirror and replicate previous published work where self-regulation factor scores derived from surveys but not tasks predicted real-world outcomes, including mental health [27]. Similarly, in a large sample of adolescents, self-reported measures of compulsivity were predictive of longitudinal developmental trajectory of a cognitive measure indexing model-based decision making. In contrast, model-based learning was not predictive of the longitudinal trajectory of symptoms [88]. Formal analytical work has further clarified why tasks might be unsuitable to capture individual differences, as, by construction, they are characterized by low between—subject variability [28, 29]. The predictive power of tasks might also have been affected by some limitations such as a relatively small sample size as well as the convenient sample recruited via MTurk. However, it is also evident that these drawbacks were not undermining the ability of survey-derived measures to predict psychiatric symptoms.

These results challenge the possibility of relying solely on existing cognitive measures to yield robust predictions for psychiatric symptoms that can be used by clinicians. One likely hypothesis is that psychiatric diseases emerge from multiple causal factors that vary across several units of analysis (i.e., molecular, social, cognitive) [89]. For example, reflecting on the ability of cognitive neuroscience in predicting real-world behavior, it has been suggested that, on analogy with genetic data where each genetic variant can account for small amount of variance, each of our (neuro)cognitive measures will have small predictive power [90]. Therefore, it might not be possible to assume the superiority of one level as the obvious and unique candidate to explain psychiatric conditions [91]. One implication is that several levels of analysis need to be embraced to reach robust predictions.

Analogous to previous interpretations, successful performance of surveys might be explained by methodological similarities between the tools used to establish the presence of psychiatric symptoms and those to evaluate self-regulation via surveys, as both rely on self-report assessment. Additionally, deciding whether a specific instrument is included among those evaluating self-regulation or psychiatric symptoms can be an arbitrary process. For example, in the original study by [27], the Barratt Impulsiveness Scale was part of the battery of surveys aimed at investigating constructs of relevance to self-regulation. However, the same questionnaire has been used to derive transdiagnostic dimensions of psychiatric symptoms [31, 32]. Here we avoided circularity by including the Barratt Impulsiveness Scale only in the set of measures to quantify transdiagnostic dimensions of psychiatry. However, it is clear that the boundaries between the two categories are labile.

Beside methodological similarities, data derived from surveys and tasks can be differentially affected by temporal influences. This aspect can influence their respective predictive abilities [92]. While questionnaires are designed to assess participant’s typical behavior averaged across long period of times, tasks tap on constructs that might potentially be influenced by transient aspects such as for example hormonal and circadian rhythm and arousal [93–95]. Interestingly, while surveys had increased predictive power compared to cognitive measures, only the latter were sensitive to environmental changes potentially associated to physiological and psychological variations. Finally, cognitive measures derived from tasks have the potential to inform on the underlying mechanisms leading to psychiatric symptoms, a possibility that is precluded to surveys.

Our results suggest that self-regulation measures obtained from surveys can be successfully leveraged to predict psychiatric symptoms. Machine learning approaches have already shown the potential for predicting response to specific antidepressant medication simply relying on self-reported symptoms obtained from questionnaires [96]. Here, we showed that transdiagnostic psychiatric dimensions exhibit both uniformity and variability. For example, although emotional control is of relevance across all psychiatric factors, an AD dimension was associated with enhanced agreeableness. In turn, a dimension related to compulsivity was related to sensation seeking and reward sensitivity. This bears on the need to effectively fractionate the components relevant for each psychiatric dimension, in order to understand its underpinning. Hence, we showed that specific self-reported measures of self-regulation, which have been associated to vulnerability and expression of psychiatric conditions [97], might represent an actionable target for prediction.

While our study employed a within-subject design, an important caveat to our conclusions is the lack of a control group not exposed to the pandemic. Secondly, this work is limited by its convenience sample recruited via Mechanical Turk. It has been shown that associations between cognitive variables and self-reported psychiatric symptoms obtained online can reflect impairments seen in patients population interviewed in person [31, 98]. However, the online nature of this study precluded face to face interview to assess symptomatology and needs further validations in clinical samples.

Overall, we used an extensive and multidimensional battery aimed at investigating self-regulation, allowing the precise dissection of orthogonal cognitive constructs relevant to successful self-regulation. Critically, a transdiagnostic analysis uncovered a relationship between specific psychiatric phenotypes and parameters linked to decision formation, which were affected concomitantly to naturally occurring stressor. Hence, we showed that cognitive functioning can change over time, possibly depending on the interaction between external events and trait-like vulnerabilities, suggesting that the study of cognition needs to take into account the dynamic nature of real-world events as well as within subject variability over time [99, 100]. Finally, our results challenge the possibility of using cognitive tasks to reach robust prediction and offer insight on different self-regulation constructs which might support the development of intervention based on multiple domains of relevance for specific psychiatric dimensions.

## Supplementary information

Supplementary Material

## Data Availability

The data underlying the analyses of this work, as well as cleaning procedures and analysis code, are available at https://github.com/MatildeVaghi/self_regulation_COVID-19. The study pre-registration is available at https://osf.io/ney9v. Deviations from pre-registration are described in the supplementary materials.
