# Supplementary material for: Relating psychiatric symptoms and self-regulation during the COVID-19 crisis
Source: Transl Psychiatry. 2022 Jul 11;12:271. doi: 10.1038/s41398-022-02030-9 (PMC9274960; doi:10.1038/s41398-022-02030-9)
Supplement: Supplementary file 1 — Supplementary Material [file 41398_2022_2030_MOESM1_ESM.docx]

**Supplementary Materials**

Some of the methods in relation to the self-regulation battery have been documented in previous work (1). For convenience we have reused part of the text from that paper in this supplement to outline details on data collection and variable selection.

**Data collection.** We aimed at identifying longitudinal changes in self-regulation putatively related to challenging real-life events connected to the emergence of the pandemic. Therefore, our participants were recruited from an existing pool of subjects (N = 522) who completed the extensive battery of cognitive tasks and surveys on self-regulation used in this study before the onset of the COVID-19 pandemic (i.e., July-September 2016) (1). To this end, we contacted 273 of the participants from the original pool who responded to recent requests of follow-up assessments (2), showing appetite for being tested again. Our data collection commenced on 5^th^ May 2020 and ended on 11^th^ June 2020 when the shelter in place order was lifted in all US states ([Wikipedia Entry](https://en.wikipedia.org/wiki/U.S._state_and_local_government_responses_to_the_COVID-19_pandemic#cite_note-109)). At the time of data collection, the pandemic was widespread and within this period US states enacted strict regulations to mitigate the spread of the virus, including limitations on social contacts as well as travelling. Upon the start of data collection, all the eligible participants were contacted and made aware of the possibility of participating in our follow-up study. Participants expressing their interest in taking part were offered the possibility of being recruited into the study. Participants were required to finish the entire battery within one week from when they started, but no other restriction was placed on their time. The majority of the participants who started the study (136 out of 145, 93.8%) completed the battery suggesting that our measures to keep attrition manageable (Supplementary Material) were effective. All participants taking part in the study confirmed their agreement filling an online informed consent form at the beginning of the study. The data analysis plan was pre-registered on the Open Science Framework (<https://osf.io/ney9v> with deviations as outlined below).

**Mechanical Turk data collection procedure.** A number of steps were taken in order to reduce attrition due to the length of our battery. Firstly, the online battery was only made available to those participants explicitly expressing their interest in our follow-up assessment. Additionally, we gave comprehensive instructions and sent follow-up emails, actively fielding questions from MTurk workers. As an incentive to complete the battery and in line with (1), we created a payment schedule that paid a lower rate if the participant failed to complete the battery. Together, these steps were successful in keeping attrition low. Of the whole sample who started the study (N = 145), only 9 participants did not complete the battery. We removed these participants as well as any who failed to pass the quality checks specified below.

**Quality check for cognitive tasks.** Participants on MTurk are wholly unsupervised, necessitating procedures to ensure data quality. Quality checks were broadly applied to all cognitive tasks to ensure that (1) response times were not unreasonably fast on average, (2) omitted responses were reasonably low, (3) accuracy on cognitive tasks was reasonably high and (4) responses were sufficiently distributed (i.e. the participant didn’t only press a single key). The specific criteria we used differed for some tasks, but in general we required that median response times were longer than 200 ms, no more than 25%​ ​of responses were omitted, accuracy was higher than 60% and no single response was given more than 95% of the time. These thresholds were determined by (1), prior to our data collection and implemented here for the new wave of data collection. Overall, these steps were taken to ensure that participants in our dataset completed the tasks in earnest. Similar checks could not be performed on the self-report surveys as we did not collect response time measures and potentially suspect response patterns (e.g., selecting only one response for every item) may be input honestly.

These criteria were used to evaluate each participant/task pair; failure on any check led to removal of that particular task’s data for that participant. In addition, we removed a participant’s entire dataset if they failed on four or more individual tasks (29 out of the 136 participants were removed). As an additional quality check, we excluded 5 participants who at the second time of testing provided information on age and on at least another demographic measure that was incompatible with that provided previously. The list of demographic measures on which participants were screened included for example “highest education achieved” or the “number of divorce counts”. On these variables, the information provided at the first testing session constrained possible successive ones.

These quality checks were intended as thresholds to screen out participants who were intentionally gaming the HIT. We also used task-specific manipulation checks which evaluated particular performance criteria specific to different tasks, necessary for the interpretability of our derived dependent measures. Failing these manipulation checks led to the removal of that participant’s data on the failed task, but did not count towards the four failed tasks that would lead to the entire participant being removed from our study.​ ​The tasks that used these additional manipulation checks were the stop signal tasks, probabilistic selection task, and two-step decision task.

**Training datasets.** For measures of self-regulation, our training dataset was composed of data from subjects who completed the battery of tasks and surveys only before the onset of the pandemic (N = 386) (Eisenberg et al., 2019). To obtain a factorial solution for transdiagnostic psychiatric dimensions, we took advantage of publicly available data (N = 497) collected by independent researchers using the same psychiatric questionnaires we included in our study (3). We used a large pool of available data (N = 2868) (4) to derive a factorial solution of the Corona Health and Impact Survey (CRISIS) aimed at investigating several aspects related to the emergence of the pandemic such as worries related to COVID-19 or the subjective impact of life changes associated to it. The training dataset was representative of the testing datasets in terms of gender, education, relationship status, divorce count as well as proportion of Hispanic/Latino included. Only a minority of subjects reported psychiatric or neurological disorders.

**Deviations from pre-registration.** Our pre-registration described the use of an empirical normal quantile transformation (QT) to allow variables to comply to a normal distribution. However, after implementing it, we recognized that this led to significant overfitting. Hence, we used a Power Transform (PT), a parametric version of a transformation to Gaussian. We used the Yeo-Johnson transform that works with positive and negative values (5) as implemented in *sklearn*. Our PT was fit to the training dataset. The estimate of the optimal parameter was then used to transform variables in the testing datasets.

For psychiatric symptoms, we used a factor analytic approach which was different from the one specified in our pre-registration. As our sample size was expected to be low, providing a relatively low subject-to-variable ratio for a de novo factor analysis for psychiatric symptoms, we originally specified the possibility of combining the training and the testing datasets of psychiatric measures to derive underlying latent structure. Instead, to be consistent with the approach adopted for self-regulation measures, we used EFA on an independent training dataset. The obtained factor solution was used to generate factor structures and fitting models for our testing dataset (see below).

As we wanted to use a two stage model (6) for visualization purposes and for equivalence testing, our linear mixed models for longitudinal analyses were implemented exactly as we specified in our pre-registration but the regressors included in the models (i.e., age, gender, and IQ) referred to values obtained during the first wave of data collection (i.e., 2016).

Finally, we tested the predictive power of self-regulation for psychiatric symptoms using data collected after the onset of the pandemic as originally specified. Additionally, we specified another model where data collected before the onset of the pandemic were used to predict psychiatric symptoms. This allowed to investigate the prospective predictive power of self-regulation for psychiatric dimensions.

**Selection of self-regulation variables.** From the 37 tasks and 22 surveys of self-regulation, we computed 204 dependent variables (DVs) identified in (1). Each survey was analyzed identically - canonical subscale scores were used as DVs. That is, items were appropriately scored (and reversed, if necessary) and summed or averaged in accordance with individual survey scoring procedures. The tasks were heterogeneous, preventing a completely generic analysis strategy. Nonetheless, many tasks involved speeded decisions between two alternatives, and are well characterized by reaction time and accuracy. It is well known that reaction time and accuracy are confounded by the speed-accuracy trade-off​, which prompted to use the drift-diffusion model (DDM) in line with (1). The basic DDM transforms accuracy and reaction time into a drift rate, threshold, and non-decision time, roughly corresponding to performance, response caution (a point along the speed-accuracy trade-off curve) and stimulus-processing/motor-planning, respectively. We fit the DDM parameters using the hierarchical DDM (HDDM). HDDM models the DDM parameters hierarchically, such that individual parameters are assumed to be drawn from a group distribution​ (7). This procedure improves data efficiency (8), and it has been shown to better capture true parameters when dealing with small datasets, or datasets corrupted by trials influenced by processes other than evidence accumulation (e.g., attentional lapses). Though individual parameter estimates are no longer independent (due to the hierarchy), hierarchical models also have been shown to improve point estimates of individual parameters, and are particularly useful when one is interested in correlations between other traits and the individual parameter estimates (9). The HDDM also allows DDM parameters to be modeled as a function of various conditions. For example, when modeling the stroop task, we modeled drift rate as a function of conflict condition while keeping the other parameters constant. Tasks that were not speeded choice tasks were heterogeneous and each analyzed according to its own scientific tradition. The full list of measures is available in Table S8 (surveys) and Table S9 (tasks).

**Data cleaning and imputation.** We implemented several steps to ensure our data met criteria for parametric analyses assuming normally distributed variables. Generally for all the self-regulation variables, we used a Yeo-Johnson power transform using *sklearn*, where the optimal parameter (i.e., fitted transformer) for stabilizing variance and minimizing skewness is estimated through maximum likelihood. Then, in line with criteria set by (1), data with values more than 2.5 times the interquartile range above the third quartile or below the first quartile, were removed. Variables that remained excessively skewed (absolute skew > 1) were dropped. Finally, as each data matrix had missing values due to our quality check procedure, we imputed the data matrix using R's missForest.

For measures of self-regulation (both tasks and survey), we estimated the fitted transformer on the training datasets and then applied it to the training and testing datasets. Outlier data were then removed. Any variable that remained excessively skewed (absolute skew > 1) after our power transform and outlier removal was dropped (training dataset: 3 DVs; testing dataset, pre onset COVID-19: 5 DVs; post onset COVID-19: 8 DVs). To ensure we did not have redundant variables in the participant-by-measure data matrix, if any two dependent variables derived from the same task or survey measure were correlated ​r​ > 0.85, one of the variables was arbitrarily removed. Using this criteria, 17 variables were dropped from the training dataset, 15 variables were dropped from the pre onset COVID-19 dataset and 8 variables were dropped from the post onset COVID datasets. This pipeline resulted in 178 DVs common to all three datasets. A minority of each data matrix was missing due to our quality check procedure (training dataset: 3.49%; pre onset COVID-19 dataset: 2.81%; post onset COVID-19 dataset: 2.0%) but these missing values were not uniformly distributed amongst the DVs. Due to additional quality control measures (manipulation checks) some tasks (the stop signal tasks, the probabilistic selection task, two-step decision task, shift task, and delay discounting titrator) resulted in DVs with substantially more missing values (between 10-30%). We imputed the data matrix using R's missForest package. Finally, for our data analysis, the total number of correct responses on the Raven’s progressive matrices was excluded from the set of DVs as we planned to use this measure as a moderator variable in a set of analyses. Similarly, the 3 DVs derived from BIS-11 were excluded because this questionnaire was part of the set of measures included to investigate individual differences in psychiatric symptoms. This resulted in a final count of 174 DVs for self-regulation (N=113 for tasks; N=61 for surveys) which were common to the three datasets and could be used for further analysis. The exact same procedure was adopted for loneliness, perceived stress, perceived social support, mindset towards stress and mindset towards the pandemic. However, as no training dataset was available, data cleaning and imputation was directly applied to the matrix of data collected in this study. No variables needed to be dropped.

**Exploratory factor analysis on the training datasets.** Recent work conducted on the set of self-regulation variables included in the current study highlighted their relative poor test-retest reliability, especially in the case of tasks (compared to surveys) (1,10). It also suggested that the challenge of obtaining robust individual differences measures can be addressed via factor scores obtained from EFA. The latter proved high stability over time which, for tasks, exceeded the reliability of the constituent variables (four-month mean test–retest reliability across factors from tasks: M = .82; surveys: M = .86; N = 150) (10). Accordingly, to base our analysis of longitudinal change on robust and reliable measures we relied on factor scores computed as follows. We applied EFA on our training datasets to generate data-driven factor structures to be applied on our testing datasets, avoiding biased estimation in subsequent analyses. Hence, for our longitudinal analyses, factor scores were computed at both testing time points making use of the weight matrix derived from EFA on the training dataset. Therefore, the same linear combination of variables was used to create factors scores for our testing datasets, represented by those participants who completed the battery twice, at different timepoints. Similarly to previous results obtained by Enkavi et al., 2019 factors scores computed at both time points using the same linear combination of constituent variables correlated highly with each other for four task factors (M = .70; min = 0.5, Perception/Response, max = 0.8, Strategic IP) and 8 survey factors (M = .83; min = 0.76, Risk Perception, max = .91, Emotional Control).

Similarly to previous results obtained on a larger sample (N = 522) (1), which included part of the data used here as a training dataset (N =386), we observed a low correlation between variables derived from tasks and surveys of self-regulation (Figure S1 A). Additionally, neither measurement set could predict variables from the other set (Figure S1 B). Therefore, based on previous and current findings, surveys and tasks variables were treated independently for separate EFAs. A parsimonious latent structure of 4 and 8 factors was identified for tasks and surveys, respectively (Figure S2). The number of factors identified diverged slightly from the original solution (1), due to our reduced sample as well as a slightly different pre-processing pipeline which resulted in fewer selected variables. For example, previous results already showed that temporal discounting variables clustered on their own (1). However, our pre-processing pipeline led to removal of those variables as they remained skewed after our PT and outlier removal (see Data Cleaning and Imputation in Supplementary Material). Therefore, as expected, the Discounting factor was not identified in our solution.

Interpretation of the obtained factor solutions for each training dataset was based on the strongest individual loadings. A summary of the highest factor loading is presented in Figure 2 and the loadings for each variables on each factor are displayed in the [online Jupyter Notebook](https://matildevaghi.github.io/self_regulation_COVID-19/sro_covid_analyses_supplementary.html). In the case of self-regulation interpretation was also informed by the correlation between the variables loadings from the factor solution obtained in the larger sample of 522 subjects (1), relating to data partially overlapping to those included in our testing dataset (N =386). Both in the case of self-regulation factors derived from tasks (Figure S3) and surveys (Figure S4), there was high concordance between individual variables loading on each factor. The only exception was represented by the Goal-Directedness and Mindfulness factors. They were independent in the original solution obtained in the bigger sample but lumped in our training dataset.

EFA solution for questionnaires of self-reported psychiatric symptoms led to the identification of the Anxious-Depression (AD), Compulsive behavior and Intrusive Thoughts (CIT), and Social Withdrawal (SW) dimensions, based on the strongest individual item loadings and in agreement with previous work (3,11). While the items from the scale aimed at measuring social-anxiety mostly loaded on the SW factor, a more heterogeneous factor structure was observed for the AD and CIT dimensions. Items from scales measuring apathy (12), depression (13) and generalised anxiety (Spielberger et al., 1983) related to an AD dimension. Instead CIT captured items from the Obsessive Compulsive Inventory Revised (OCI-R) (14), the Barratt Impulsiveness Scale (BIS-11) (15), and the Eating Attitude Test (EAT-26) (16), which are employed to measure out of control behaviors, traditionally characterizing disorders of compulsivity.

10 factors were identified by EFA on the CRISIS questionnaire (Nikolaidis et al., 2021). Questions on daily behaviors clustered in separate factors related to Media usage, Physical exercise, Sleep Hours, Sleep time. Questions related to life changes were separated into a factor capturing the Stress induced by life changes due to the pandemic, Changes in relationship and Economic concern. Finally a set of factors related to the emotional responses to the pandemic captured General anxiety, Negative Mood, and COVID-19 worries (Figure 2). These factor scores were used in the context of linear models aimed at investigating the role of demographic characteristics (i.e., age and gender), psychiatric symptoms, and mindset attitudes towards aspects related to the COVID-19 pandemic. *lme4* (17) packages in R was used and significance values were FDR corrected over the number of the dependent variables tested (N = 10).

**Longitudinal analysis.** Our linear mixed models used factor scores on each of the task (N = 4) and survey (N = 8) self-regulation factors as dependent variables. For each factor, each subject had a value that related to data collected before or after the onset of the pandemic. By including the interaction with Time *_pre/post covid_* the models tested if each of the psychiatric factor related to the amount of longitudinal changes in self-regulation, while controlling for age, IQ, and gender (coded as male: 0; female: 1). Significance values were FDR corrected over the number of dependent variables tested within each set of models (factors from self-regulation tasks N = 4; factors from self-regulation surveys N = 8). In the syntax of the *lmer* function in R, the regressions were:

*Self-regulation* _i_ ~

*(Age + Gender + IQ + Psych _AD_ + Psych _CIT_ + Psych _SW_ )* Time _pre/post covid_ + (1|Subj)*

All models successfully converged and QQ plots were inspected for each of them. To test the robustness of our findings and in order to ascertain that results were not driven by influential observations, we used the *influence.ME* package in R (18), providing tests for detecting whether single cases affect the level of significance of an estimate in generalized mixed effects models. Accordingly, each of our model was estimated and *influence* was used subsequently to obtain information on how removing observations iteratively changed the parameter estimates. Then, we used *cook.distance* to calculate standardized measures of influence. An observation with a Cook’s Distance larger than three times the mean Cook’s Distance was regarded as an influential case, in line with common recommendations. All the results deemed significant in our original models and presented in the manuscript were unaffected by the exclusion of influential cases.

When the results of the interactions were statistically significant, we also evaluated changes in self-regulation using an analysis of covariance conditioning on baseline to confirm that our results were not due to the specific analytical approach adopted. In this set of analyses, we used the self-regulation factor score following the onset of COVID-19 as dependent variable with the baseline measurement (i.e., self-regulation before the onset of COVID-19) as covariate together with Age, Gender, IQ and each of the psychiatric factors (19).

In order to be able to declare absence of meaningful effects of the interactions included in our models we applied equivalence testing (20,21). Our outcome measures did not contain any missing data for any subjects, hence a two stage model can be used to produce the results of the mixed models (6). To improve interpretability of the equivalence test measure, we used this two stage approach by computing a paired difference for the dependent variable of interest of each model, and calculating its partial correlation with the regressors of interest. The interpretation of these partial correlations and their inferences match that of the interaction terms in the mixed model. Hence, we calculated the 90% CI using a standard Fisher’s Z transformation based CI on the estimated partial correlations. If the 90% CI was included within the boundaries for the medium cut off (i.e., -0.3, 0.3) (22), the effect was deemed smaller than the smallest effect of interest, hence equivalent to a null result (Figure S6-S8).

In a separate set of models, the dependent variables were represented by the self-reported scores of perceived stress, loneliness, and reduced social support, with reference to period preceding or following the onset of the pandemic. The models were equivalent to the ones specified for self-regulation but mindset towards stress and on whether the pandemic was subjectively perceived as a catastrophe were included to ask if they affected longitudinal changes on wellbeing measures. Significance values were FDR corrected over the number of dependent variables tested (N = 3).

*Wellbeing _i_ ~*

*(Mindset stress + Mindset pandemic + Psych _AD_ + Psych _CIT_ + Psych _SW_ + Age + Gender )* Time _pre/post covid_ + (1|Subj)*

**Prediction analyses.** As we identified a relationship between transdiagnostic dimensions of psychopathology and measures of self-regulation, we sought to understand if factor scores from tasks or surveys could be used as features to predict psychiatric symptoms. Separate analyses were conducted to evaluate the perspective or cross-sectional power of self-regulation by using task and survey factor scores referring to the period preceding or following the onset of COVID-19, respectively. In the primary prediction analyses, transdiagnostic factors of psychopathology were predicted. Complementary analyses were conducted by using the individual variables obtained from each separate questionnaire investigating psychiatric symptoms as targets of the prediction analysis. Prior to running the prediction analysis, a linear regression was used to regress age, gender and IQ out of each of predicted variables, being either a transdiagnostic factor or an individual variable of psychopathology. We used a ridge regression, availing of *scikit-learn* with built-in cross-validation capabilities to select the best hyper-parameter. Ridge regression was preferred over lasso due to its desirable regularization properties. In fact sparse feature selection is not necessary for interpretability with so few predictors. Additionally, interpretability of feature weights was improved due to the low correlation between the features included in the model. We used both insample and cross-validated prediction, using the same fitting models. For insample predictions, the models were fitted to the whole dataset and tested on the same dataset. In contrast, cross-validation was performed using a balanced 10-fold procedure, fitting each model with 91 or 92 participants and testing on the 11 or 10 left out participants. The insample and cross-validated predictions were correlated with the actual target variables (i.e., transdiagnostic factor scores or individual variables from psychiatric questionnaires) to obtain R^2^. Mean Absolute Error (MAE) was computed analogously. Cross-validated and insample predictions are shown in Tables S9 - S10. We also created an empirical null distribution of prediction success by shuffling the target outcomes and repeating the prediction 2500 times. 95% prediction success is shown in all prediction plots and is used as a significance cut off (P < .05).

The same procedure was adopted to test if self-regulation could predict changes in health risk behavior. In this additional prediction analysis, tasks and survey factor scores from the period preceding the onset of COVID-19 were used as features to predict change in health risk behavior from the from the period preceding the onset of COVID-19 to the one following it. A description of how change in health risk behavior was computed is provided in the Supplementary Methods.

**Change in health risk behavior.** In a secondary prediction analysis we tested whether self-regulation factors referring to the period preceding the onset of the pandemic could predict changes in health risk behavior. Accordingly, we derived measures of health risk behavior following identical procedures devised for surveys and tasks for self-regulation. Namely, we applied EFA on our training dataset, composed of data on health risk behavior from subjects who answered those questions only before the onset of the pandemic (N = 386) (Eisenberg et al., 2019). The list of the individual items included can be found in Table S13 and was composed of surveys designed by others to assess particular health-related behavior and a set of items devised ad hoc. Before running EFA age and gender were regressed out of each health risk behavior variable. Overall model fit was satisfactory (R^2^ = .38; RMSEA = .052; RMSR = .048). The Bayesian Information Criteria yielded 6 factors: Binge Drinking, Daily Smoking, Drug Use, Lifetime smoking, Mental Health, Problem Drinking (See Figure S10 for the factor scores correlation and the [*online Jupyter Notebook*](https://matildevaghi.github.io/self_regulation_COVID-19/sro_covid_analyses_supplementary.html) for the full factor loading matrix). Then, via *predict* from the psych package in R, the same linear combination of variables was used to create factors scores for our testing datasets, represented by those participants who completed the battery twice, at different timepoints. Hence, for each subject, we obtained measures of health risk behavior of the period preceding and following the onset of the pandemic. Measures of change were obtained by regressing health risk behavior pre COVID-19 (e.g., Mental Health _pre COVID-19)_ from the health risk behavior following COVID-19 (e.g., Mental Health _outcome COVID-19_). Each of the obtained measures represented changes in health risk behaviors and were used as dependent variables of the prediction analysis described in the Material and Methods.

**SUPPLEMENTARY FIGURES AND LEGENDS**


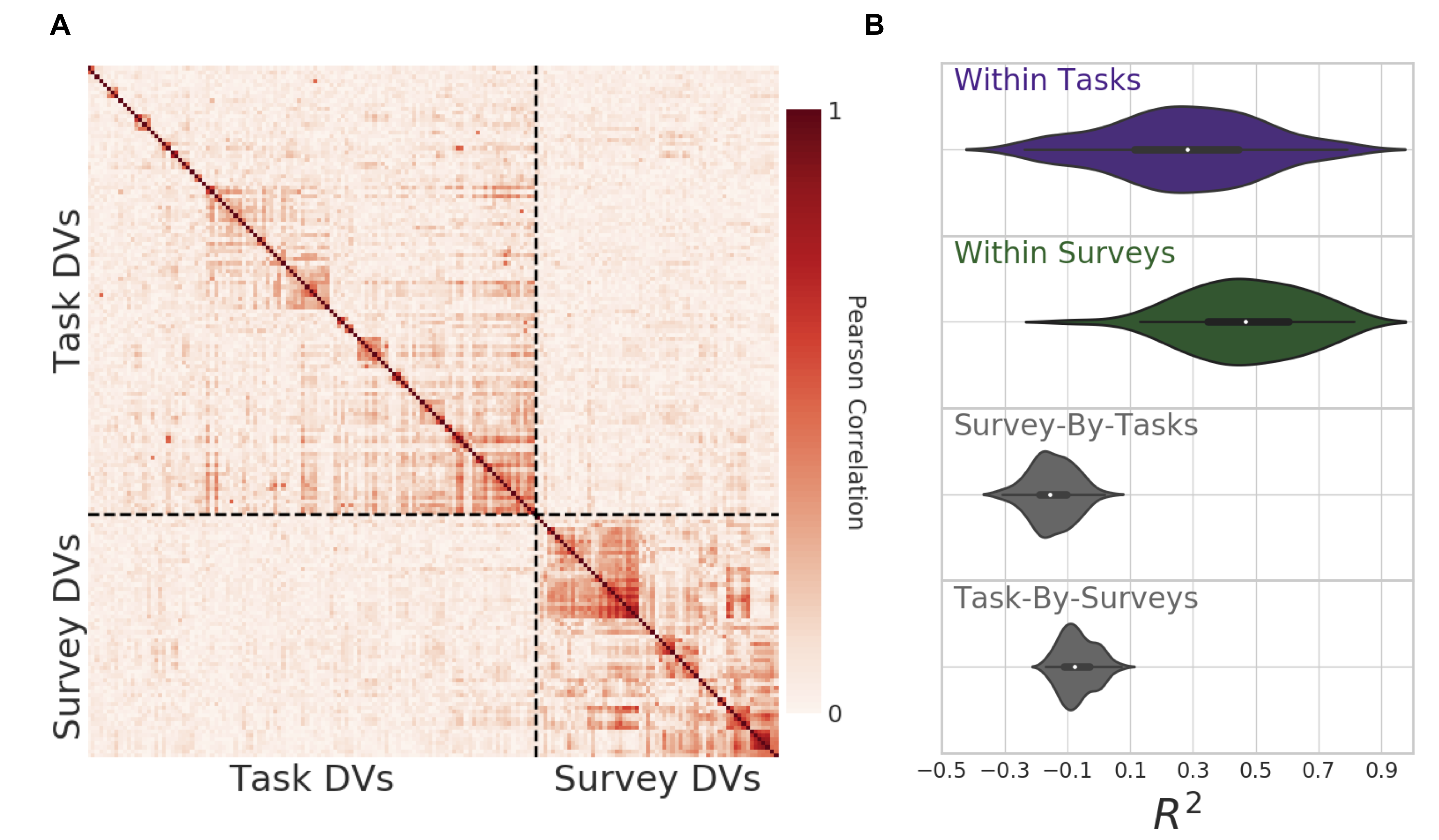


***Figure S1.*** *Relationship between variables of self-regulation on the training dataset (N=386) (A) Pearson correlation for variables of self-regulation. Variables are organized by category (i.e., tasks, surveys) and ordered based on the respective hierarchical clustering solutions. (B) Cross-validated R^2^ derived from cross-validated ridge regression of either a single task or survey variable using all survey or task variables (holding out the target).*


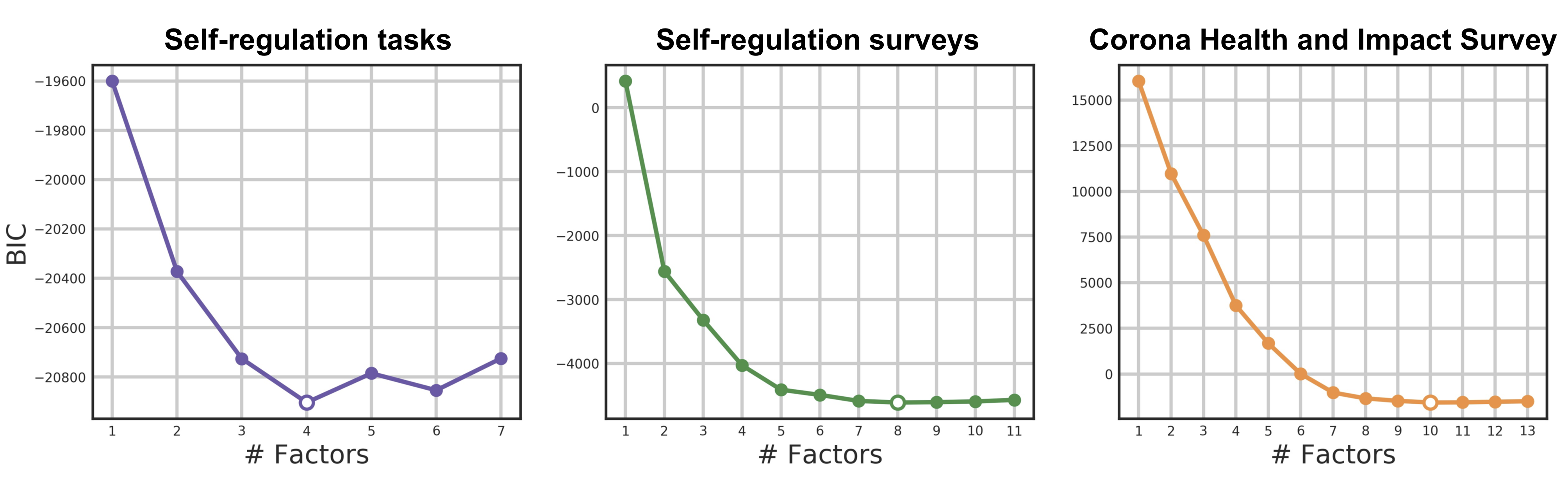


***Figure S2.*** *Bayesian information criterion (BIC) curves for EFA conducted on the training datasets. The BIC values for a range of factors are shown for EFA on variables from the self-regulation tasks, surveys and from the Corona Health and Impact Survey (CRISIS). An optimal dimensionality of 4, 8, and 10 factors (empty circle in the plot) was identified for tasks, surveys, and the Corona Health and Impact Survey respectively.*

*
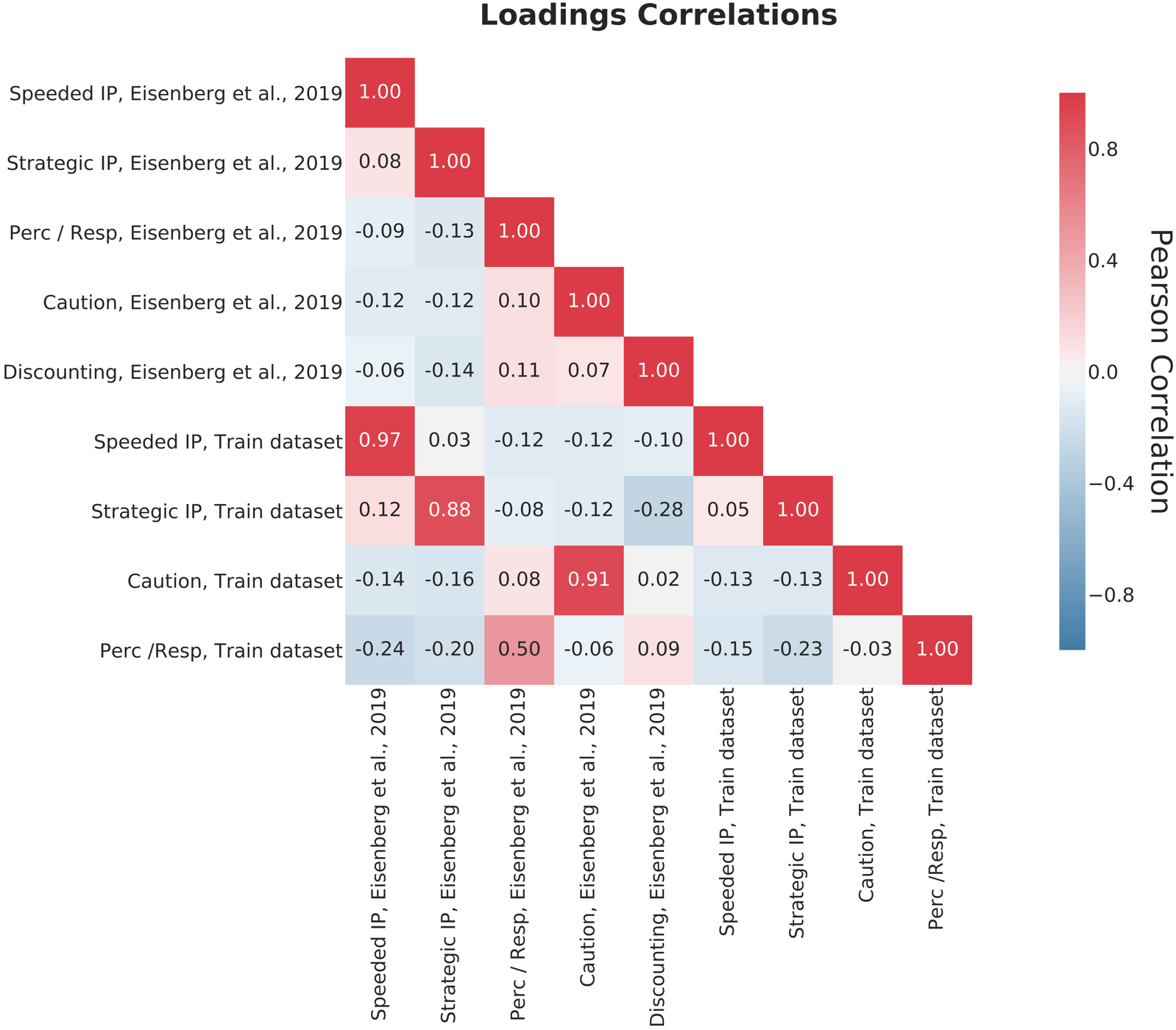
*

***Figure S3.*** *The heatmap displays the correlation between the self-regulation task variables loading for each of the factors obtained from EFA on the larger sample of 522 subjects (1) and that on the subset of participants of participants used as a training dataset in the current study (N = 386). IP, Information Processing; Perc /Resp, Perception/Response.*

***
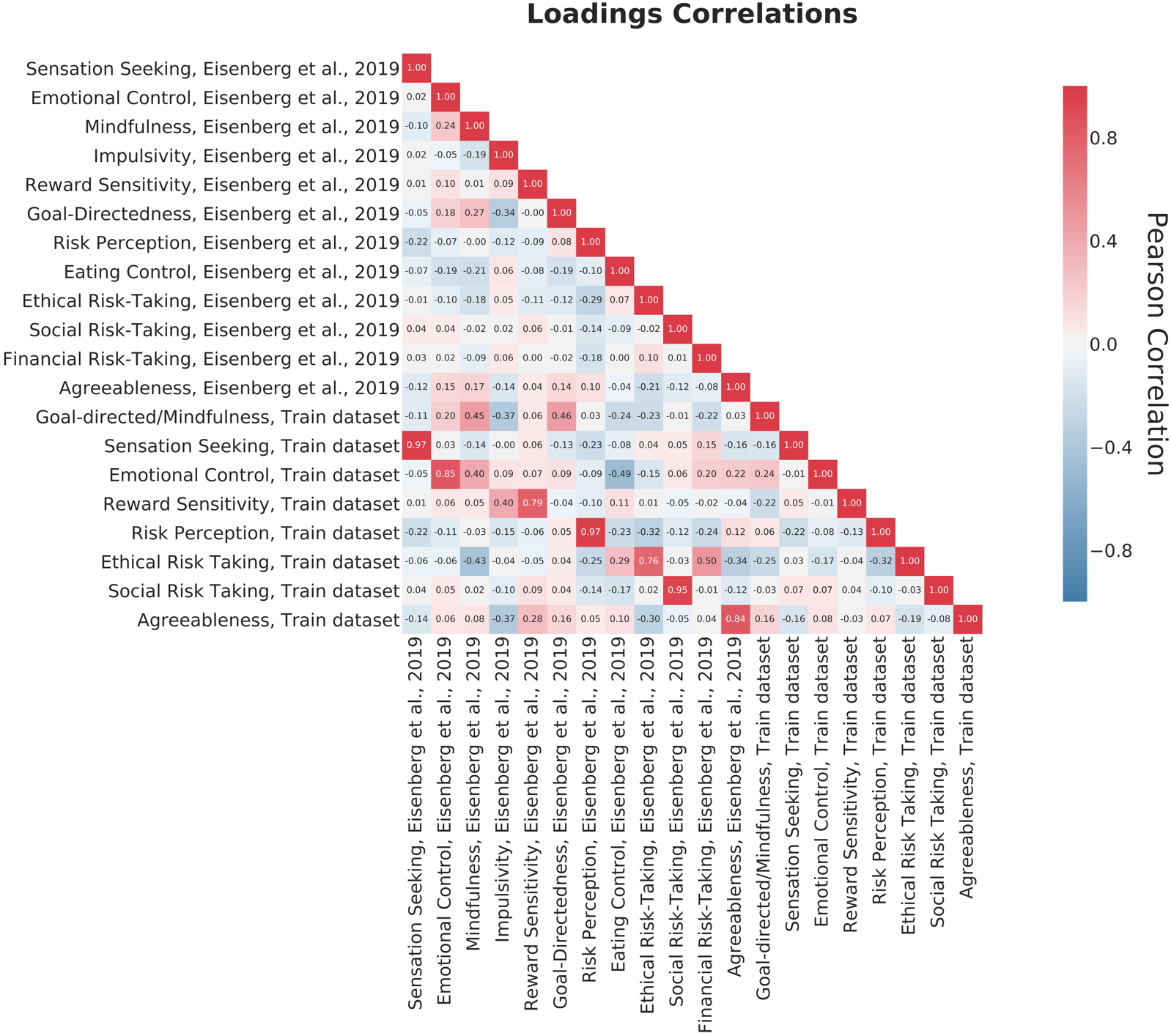
***

***Figure S4.*** *The heatmap displays the correlation between the self-regulation survey variables loading for each of the factors obtained from EFA on the larger sample of 522 subjects (1) and that on the subset of participants of participants used as a training dataset in the current study (N = 386).*

*
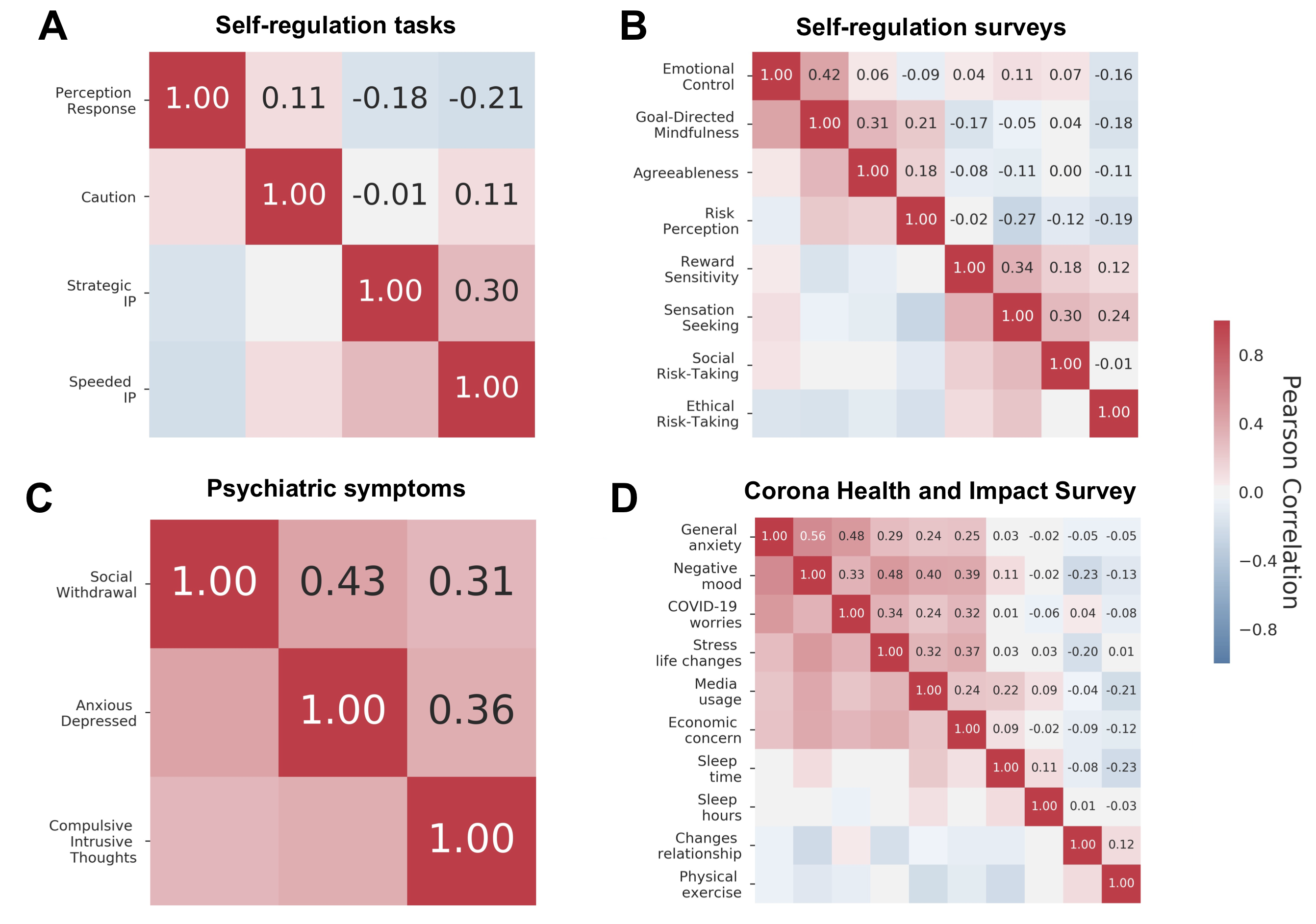
*

***Figure S5.*** *Correlation of factor scores from Exploratory Factor Analysis models on the training datasets. Factor analysis on self-regulation variables from (A) tasks and (B) surveys revealed a 4 and 8 factor solution, respectively. (C) A 3 factor solution was obtained on the training dataset of psychiatric symptoms. (D) A 10 factor solution was derived from the Corona Health and Impact Survey (CRISIS). The heatmaps display the moderate correlations (r Pearson’s correlation < .05) between different factors, reflecting their relative independence.*


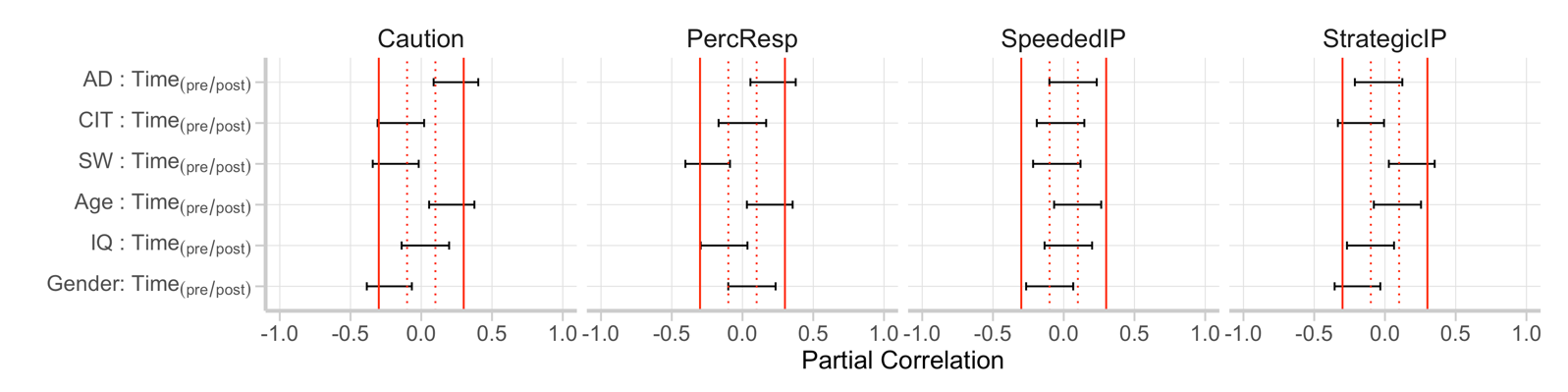


***Figure S6. Equivalence testing for models testing longitudinal changes in task-based self-regulation.*** *We computed a paired difference for the dependent variable of interest (e.g., Speeded IP _post onset COVID-19_ – Speeded IP _pre onset COVID-19_) of each model and calculated its partial correlation with the regressors included in the model (e.g., AD, CIT, SW, Age, IQ, Gender). The plot reflects the partial correlation obtained and the associated 90 % CI, computed using a standard Fisher’s Z transformation based. The results are effectively a test of equivalence for the interactions included in the mixed models (Material and Methods) reported in Table S4, hence the labels on the y axis. Dotted and continuous line correspond to a small (i.e., -0.1, 0.1) and medium (i.e., -0.3, 0.3) cut off respectively. When the 90% CI was included within the boundaries for the medium cut off (i.e., -0.3, 0.3), the effect was deemed smaller than the smallest effect of interest, hence equivalent to a null result. AD, Anxious-Depression; CIT, Compulsive behavior and Intrusive Thoughts; SW, Social Withdrawal.*


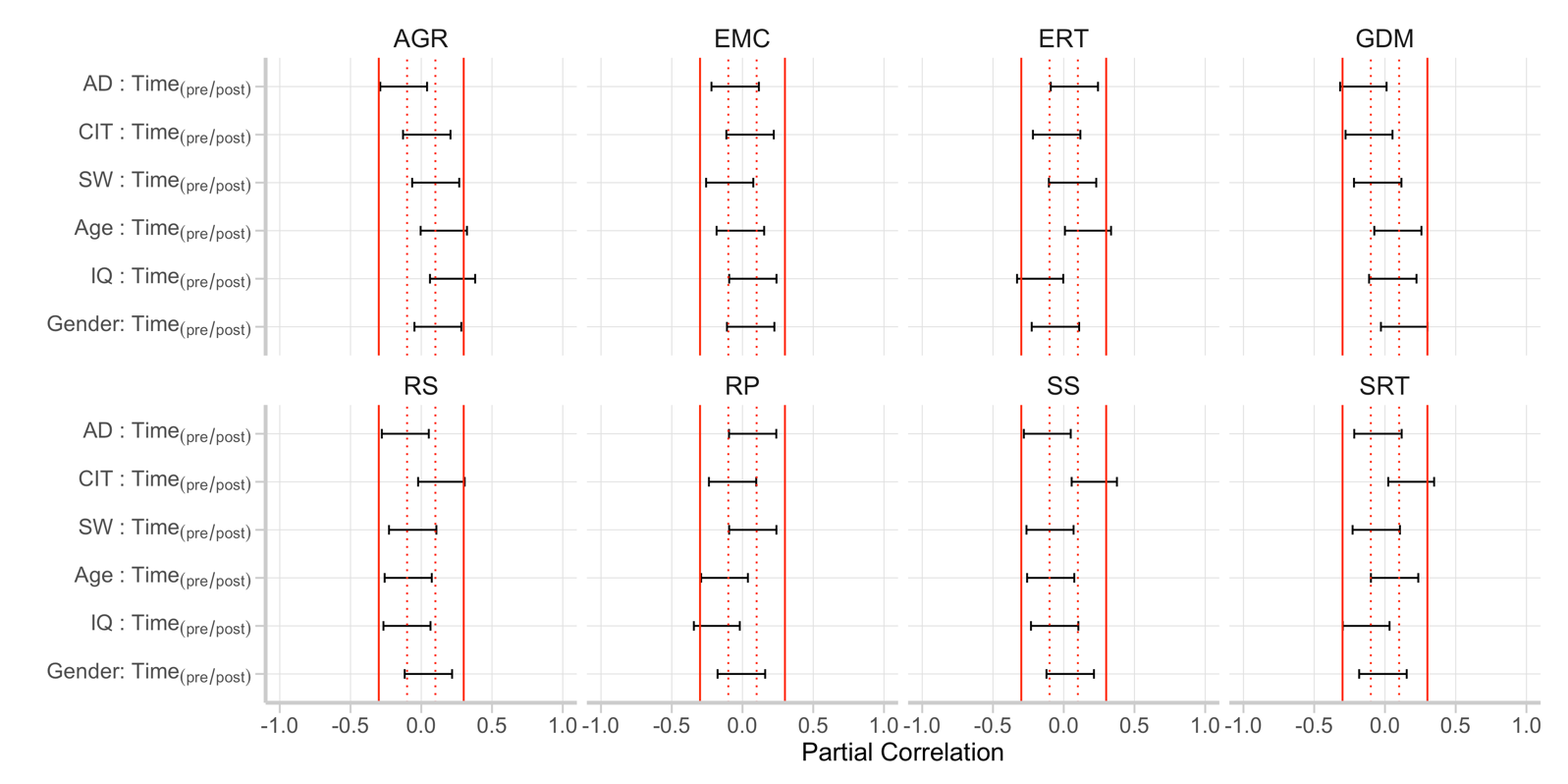


***Figure S7. Equivalence testing for models testing longitudinal changes in survey-based self-regulation.*** *We computed a paired difference for the dependent variable of interest (e.g., EMC _post onset COVID-19_ – EMC _pre onset COVID-19_) of each model and calculated its partial correlation with the regressors included in the model (e.g., AD, CIT, SW, Age, IQ, Gender). The plot reflects the partial correlation obtained and the associated 90 % CI, computed using a standard Fisher’s Z transformation based. The results are effectively a test of equivalence for the interactions included in the mixed models (Material and Methods) reported in Table S5, hence the labels on the y axis. Dotted and continuous line correspond to a small (i.e., -0.1, 0.1) and medium (i.e., -0.3, 0.3) cut off respectively. When the 90% CI was included within the boundaries for the medium cut off (i.e., -0.3, 0.3), the effect was deemed smaller than the smallest effect of interest, hence equivalent to a null result. AD, Anxious-Depression; CIT, Compulsive behavior and Intrusive Thoughts; SW, Social Withdrawal. AGR, Agreeableness; EMC, Emotional Control; ERT, Ethical Risk-Taking; GDM, Goal-Directed/Mindfulness; RS, Reward Sensitivity; RP, Risk Perception; SS, Sensation Seeking; SRT, Social Risk-Taking.*


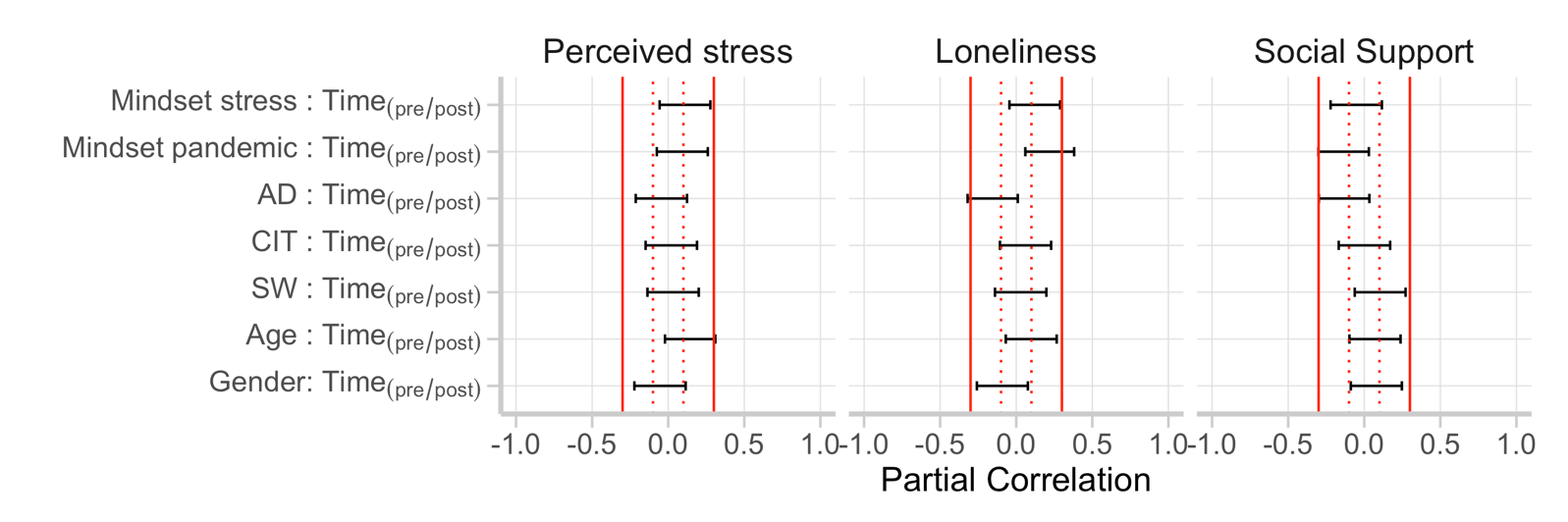


***Figure S8. Equivalence testing for models examining trajectories of well-being related to the pandemic onset.*** *We computed a paired difference for the dependent variable of interest (e.g., Perceived stress _post onset COVID-19_ – Perceived stress _pre onset COVID-19_) of each model and calculated its partial correlation with the regressors included in the model (e.g., mindset stress, mindset pandemic, AD, CIT, SW, Age, Gender). The plot reflects the partial correlation obtained and the associated 90 % CI, computed using a standard Fisher’s Z transformation based. The results are effectively a test of equivalence for the interactions included in the mixed models (Material and Methods) reported in Table S3, hence the labels on the y axis. Dotted and continuous line correspond to a small (i.e., -0.1, 0.1) and medium (i.e., -0.3, 0.3) cut off respectively. When the 90% CI was included within the boundaries for the medium cut off (i.e., -0.3, 0.3), the effect was deemed smaller than the smallest effect of interest, hence equivalent to a null result. AD, Anxious-Depression; CIT, Compulsive behavior and Intrusive Thoughts; SW, Social Withdrawal.*


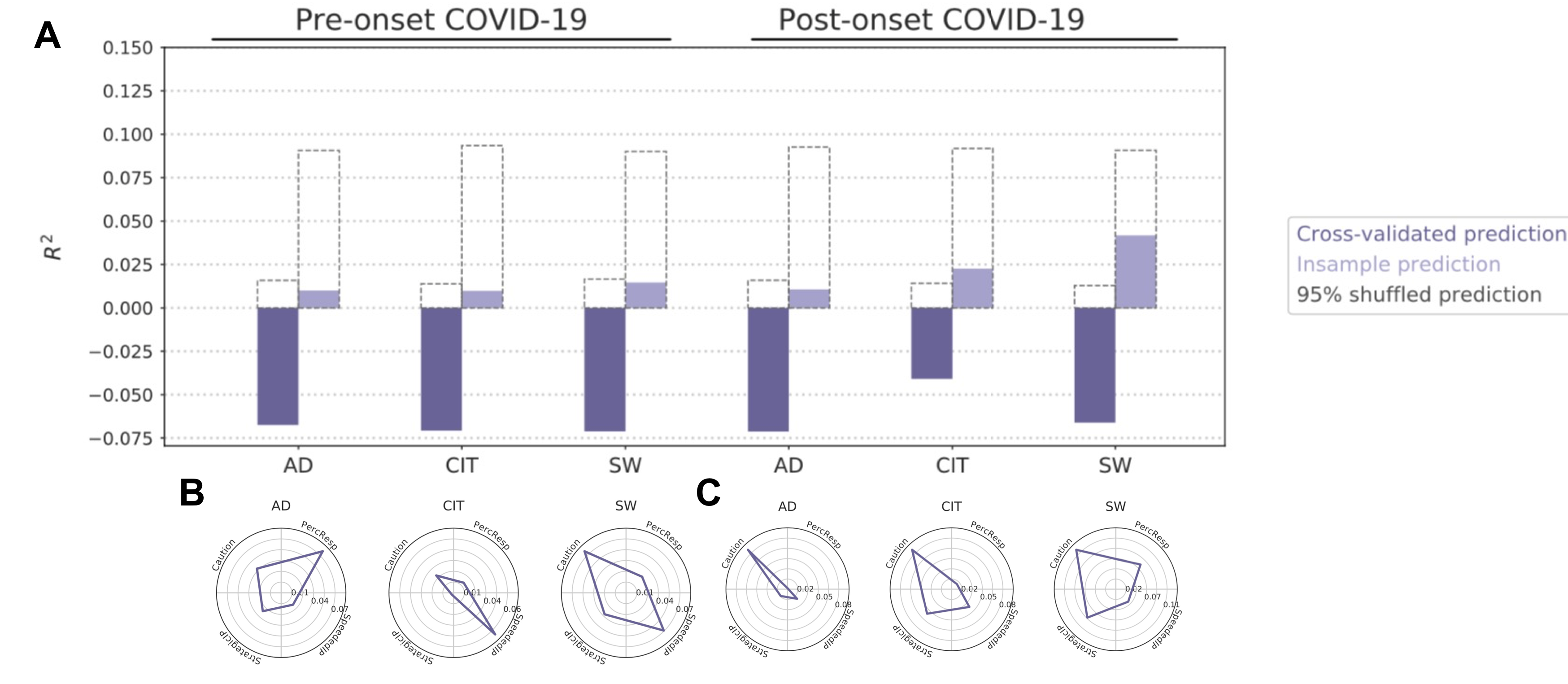


***Figure S9.*** *Prediction of psychiatric symptoms dimension using task factor-scores. (A) Predictions where self-regulation factors scores of the period preceding (pre, prospective) or following (post, cross-sectional) the onset of COVID-19 were used to predict psychiatric symptoms assessed during the emerging of the COVID-19 pandemic. In the bottom row, fingerprints using factor scores of the period preceding (B) or following (C) the onset of the COVID-10 pandemic are displayed. Dark and light bars indicate R^2^ cross-validated and insample prediction respectively. Dashed gray boxes indicate 95% of null distribution, estimated from 2500 shuffles of the target outcome. Fingerprints displayed as polar plots indicate the standardized β for each factor. Perc/Resp, Perception/Response; IP, Information Processing; AD, Anxious-Depression; CIT, Compulsive behavior and Intrusive Thoughts; SW, Social Withdrawal.*

**
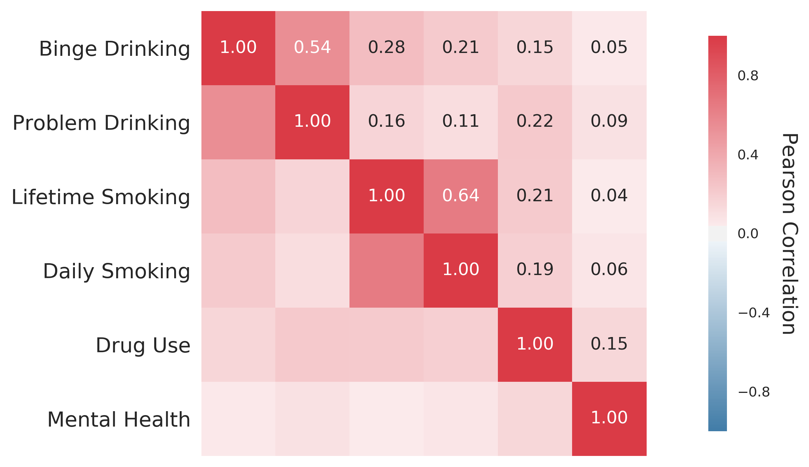
**

***Figure S10.*** *Correlation of factor scores from Exploratory Factor Analysis models on the training dataset for health risk behavior measures. Pearson correlation amongst factors is displayed.*

**
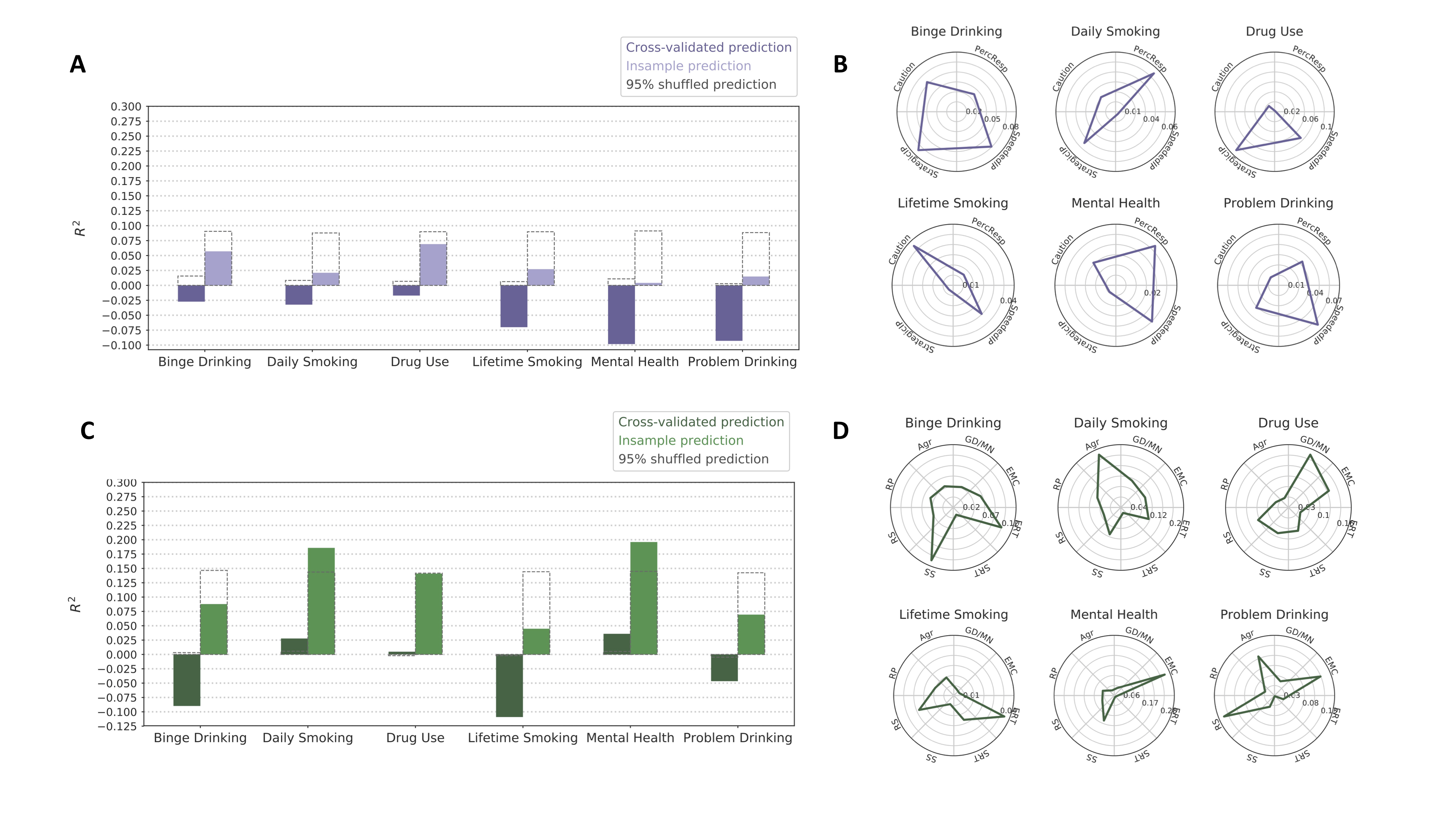
**

***Figure S11.*** *Prediction of change in health risk behaviors. (A) Tasks- and (C) survey-related self-regulation factors scores of the period preceding the onset of COVID-19 were used to predict change in health risk behaviors. Fingerprint corresponding to (B) tasks and (D) survey factor scores. Dark and light bars indicate R^2^ cross-validated and insample prediction respectively. Dashed gray boxes indicate 95% of null distribution, estimated from 2500 shuffles of the target outcome. Fingerprints displayed as polar plots indicate the standardized β for each factor. The y-axes are scaled for each fingerprint to highlight the distribution of associations—no inference can be drawn comparing individual factor magnitudes across outcomes. EMC, Emotional Control; GD/MND, Goal-Directed/Mindfulness; Agr, Agreeableness; RP, Risk Perception; RS, Reward Sensitivity; SS, Sensation Seeking; SRT, Social-Risk Taking; ERT, Ethical Risk-Taking.*

**
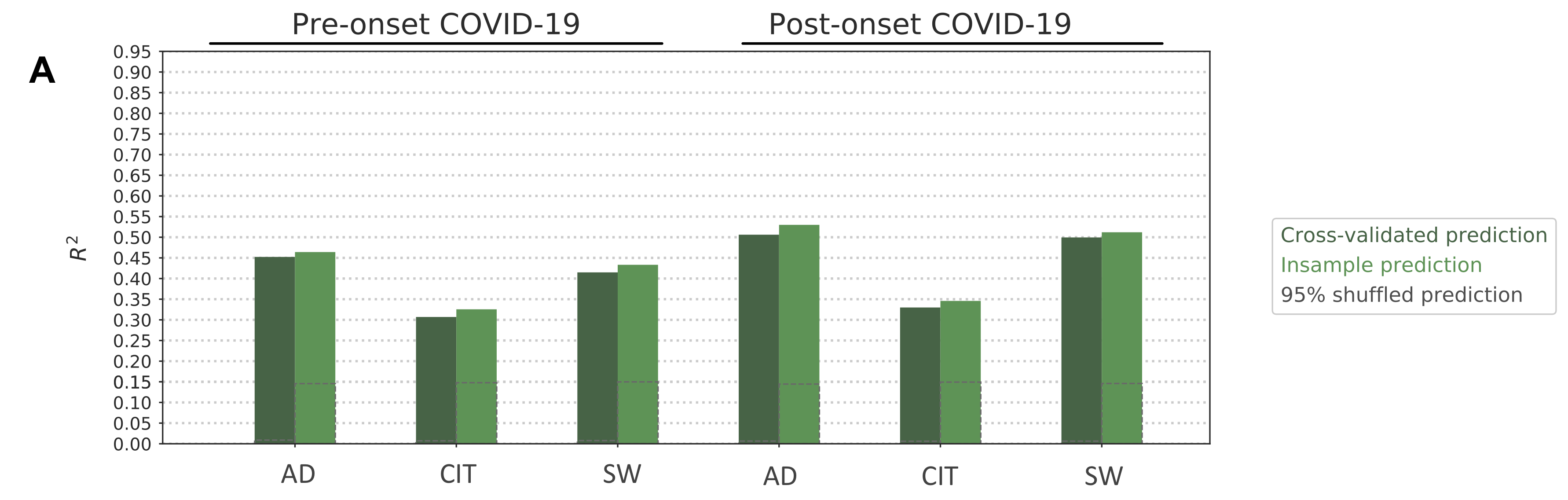
**

***Figure S12.*** *Prediction of psychiatric symptoms dimension using a reduced regression model including only Emotional Control survey factor-score as predictor. (A) Results of the reduced regression model where Emotional Control survey-factor scores of the period preceding (pre, prospective) or following (post, cross-sectional) the onset of COVID-19 was used to predict psychiatric dimensions assessed during the initial phase of the COVID-19 pandemic. Dark and light bars indicate R^2^ cross-validated and insample prediction respectively. Dashed gray boxes indicate 95% of null distribution, estimated from 2500 shuffles of the target outcome. AD, Anxious-Depression; CIT, Compulsive behavior and Intrusive Thoughts; SW, Social Withdrawal.*

**
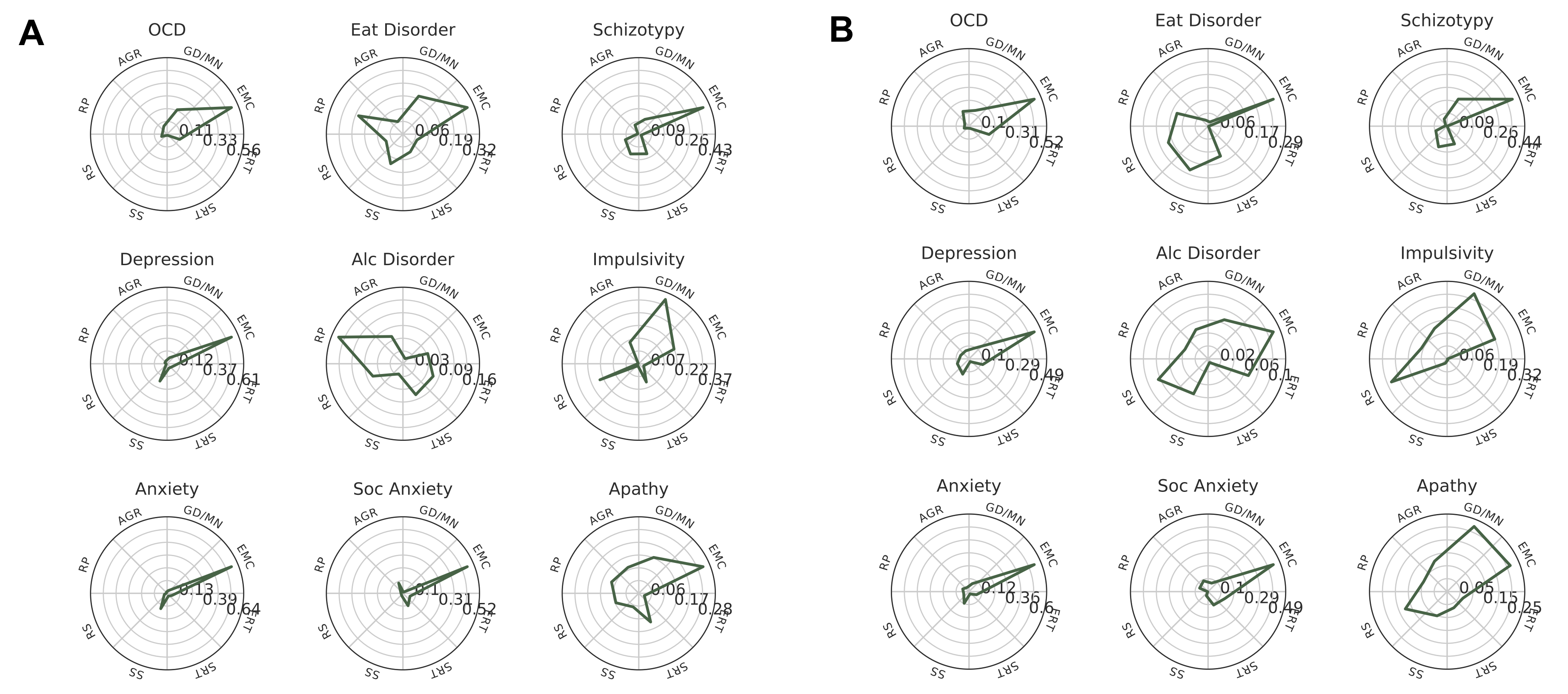
**

***Figure S13.*** *Prediction of individual psychiatric variables using survey factor scores of the period preceding (A) or following (B) the onset of the COVID-10 pandemic. Fingerprints displayed as polar plots indicate the standardized β for each factor. The y-axes are scaled for each fingerprint to highlight the distribution of associations—no inference can be drawn comparing individual factor magnitudes across outcomes. These plots are the equivalent of those displayed in Figure 5 in relation to the psychiatric transdiagnostic measures. EMC, Emotional Control; GD/MND, Goal-Directed/Mindfulness; Agr, Agreeableness; RP, Risk Perception; RS, Reward Sensitivity; SS, Sensation Seeking; SRT, Social-Risk Taking; ERT, Ethical Risk-Taking.*

**Table S1.** Demographic characteristics of subjects included in the training and testing datasets for self-regulation

|  |  | **Training**  **dataset**  (N= 386) | | **Pre-onset**  **COVID-19**  (N = 102) | | **Post-onset**  **COVID-19**  (N = 102) | | |
| --- | --- | --- | --- | --- | --- | --- | --- | --- |
| **Age** | Mean  (SD) | 32.46  (7.51) | | 36.61  (7.86) | | 40.26  (7.80) | | |
| **Gender*** | Female | 194 (50.26%) | | 50 (49.02%) | | 49 (48.04%) | | |
|  | Male | 192 (49.74%) | | 52 (50.98%) | | 52 (50.98%) | | |
|  | Non-binary/ Third gender |  | |  | | 1 (0.98%) | | |
| **Hispanic/Latino** | No | 359 (93.01%) | | 94 (92.16%) | | 94 (92.16%) | | |
|  | Yes | 27 (6.99%) | | 8 (7.84%) | | 8 (7.84%) | | |
| **Highest Education** | Advanced Graduate work or Ph.D | 9 (2.33%) | | 4 (3.92%) | | 3 (2.94%) | | |
|  | Bachelor's Degree | 147 (38.08%) | | 38 (37.25%) | | 44 (43.14%) | | |
|  | High School/GED | 52 (13.47%) | | 16 (15.69%) | | 14 (13.73%) | | |
|  | Master's Degree | 15 (3.89%) | | 6 (5.88%) | | 7 (6.86%) | | |
|  | Some College | 160 (41.45%) | | 38 (37.25%) | | 34 (33.33%) | | |
|  | Did Not Complete High School | 3 (0.78%) | |  | |  | | |
| **Relationship Status** | Committed Relationship | 96 (24.87%) | | 24 (23.53%) | | 19 (18.63%) | | |
|  | Married | 124 (32.12%) | | 35 (34.31%) | | 38 (37.25%) | | |
|  | Single | 166 (43.01%) | | 43 (42.16%) | | 45 (44.12%) | | |
| **Divorce Count** | 0 | 339 (87.82%) | | 88 (86.27%) | | 87 (85.29%) | | |
|  | 1 | 40 (10.36%) | | 11 (10.78%) | | 13 (12.75%) | | |
|  | 2 | 6 (1.55%) | | 3 (2.94%) | | 2 (1.96%) | | |
|  | 3 | 1 (0.26%) | |  | |  | | |
| **Psychiatric disorder** | Attention Deficit Hyperactivity Disorder | 14 (3.63%) | | 3 (2.94%) | | 6 (5.88%) | | |
|  | Alcohol Dependency | 3 (0.78%) | | 3 (2.94%) | | 3 (2.94%) | | |
|  | Anorexia Nervosa | 6 (1.55%) | | 1 (0.98%) | | 1 (0.98%) | | |
|  | Anxiety Disorder | 66 (17.10%) | | 16 (15.69%) | | 22 (21.57%) | | |
|  | Borderline Personality Disorder | 1 (0.26%) | |  | | 1 (0.98%) | | |
|  | Depression | 32 (8.29%) | | 7 (6.86%) | | 4 (3.92%) | | |
|  | Drug Dependency | 3 (0.78%) | | 1 (0.98%) | | 1 (0.98%) | | |
|  | Manic-Depressive (Bipolar) illness | 2 (0.52%) | | 1 (0.98%) | |  | | |
|  | Obsessive Compulsive Disorder | 3 (0.78%) | |  | | 1 (0.98%) | | |
|  | Other | 8 (2.07%) | | 3 (2.94%) | | 1 (0.98%) | | |
|  | None | 248 (64.25%) | | 67 (65.69%) | | 62 (60.78%) | | |
| **Neurological disorder** | Yes | | 5 (1.30%) | | 2 (1.96%) | | 2 (1.96%) |  |
|  | No | | 381 (98.70%) | | 100 (98.04%) | | 100 (98.04%) |  |

**The question about gender was modified when collecting data after the onset of COVID-19, to account for non-binary or third gender; SD, Standard Deviation. Unless otherwise specified, number of subjects and associated percentage for the given category are shown in the table.*

**Table S2.** Summary of model fit measures for each Exploratory Factor Analysis on the training datasets

|  | **N Subjects** | **N Factors** | **R^2^** | **RMSEA** | **RMSR** |
| --- | --- | --- | --- | --- | --- |
| **EFA on tasks of self-regulation** | 386 | 4 | .21 | .060 | .060 |
| **EFA on surveys of self-regulation** | 386 | 8 | .51 | .064 | .039 |
| **EFA on self-reported psychiatric symptoms** | 497 | 3 | .27 | .044 | .058 |
| **EFA on the Corona Health and Impact Survey** | 2868 | 10 | .48 | .029 | .014 |

*EFA, Exploratory Factor Analysis; BIC, Bayesian Information Criterion; RMSEA, Root Mean Square Error of Approximation; RMSR, Root Mean Square of the Residuals. The RMSR is and empirical value and it is comparable to the RMSEA, which is based upon normal theory and the non-central χ^2^. A RMSEA <.08 is indicative of a good fit (23).*

**Table S3.** Results from linear mixed models examining longitudinal changes in task-related measures of self-regulation

|  | **Caution** | | | **Perception/ Response** | | | **Speeded IP** | | | **Strategic IP** | | |
| --- | --- | --- | --- | --- | --- | --- | --- | --- | --- | --- | --- | --- |
|  | **β**  **(SE)** | **t value** | **p value**  **[95% CI]** | **β**  **(SE)** | **t value** | **p value**  **[95% CI]** | **β**  **(SE)** | **t value** | **p value**  **[95% CI]** | **β**  **(SE)** | **t value** | **p value**  **[95% CI]** |
| Intercept | -.05  (.09) | -.59 | .56  [-.23, .12] | -.05  (.08) | -.66 | .51  [-.20, .10] | -.03  (.07) | -.34 | .73  [-.17, .12] | .02  (.07) | .34 | .73  [-.10, .15] |
| AD | .25  (.12) | 1.99 | .05  [.01, .48] | -.02  (.11) | -.2 | .84  [-.23, .19] | .03  (.1) | .31 | .76  [-.16, .22] | .07  (.09) | .81 | .42  [-.10, .25] |
| CIT | -.12  (.11) | -1.1 | .27  [-.34, .09] | .06  (.10) | .63 | .53  [-.13, .25] | -.16  (.09) | -1.71 | .09  [-.33, .02] | -.17  (.08) | -2.08 | .04  [-.33, .01] |
| SW | -.19  (.12) | -1.61 | .11  [-.42, .04] | -.06  (.10) | -.6 | .54  [-.26, .14] | .09  (.1) | .98 | .33  [-.09, .28] | .1  (.09) | 1.11 | .27  [-.07, .27] |
| Age | **.24**  **(.09)** | **2.53** | **.01***  **[.06, .42]** | .14  (.08) | 1.6 | .11  [-.03, .29] | .13  (.08) | 1.69 | .01  [-.02, .28] | **-.24**  **(.07)** | **-3.38** | **<.001*****  **[-.37, -.10]** |
| IQ | .05  (.09) | .58 | .56  [-.12, .23] | -.11  (.08) | -1.32 | .19  [-.26, .05] | **.33**  **(.08)** | **4.5** | **<.001*****  **[.19, .48]** | **.56**  **(.07)** | **8.21** | **<.001*****  **[.43, .69]** |
| Gender | -.08  (.09) | -.91 | .37  [-.26, .09] | 0  (.08) | -.05 | .96  [-.16, .15] | .02  (.08) | .3 | .77  [-.12, .17] | -.14  (.07) | -2.03 | .05  [-.27, -.01] |
| Time _pre/post_ | .01  (.07) | .11 | .91  [-.12, .13] | -.03  (.09) | -.3 | .77  [-.20, .14] | .01  (.06) | .12 | .91  [-.11, .13] | -.02  (.06) | -.29 | .77  [-.13, .10] |
| AD:  Time _pre/post_ | **.23**  **(.09)** | **2.53** | **.01***  **[.06, .40]** | .27  (.12) | 2.24 | .03  [.04, .50] | .06  (.09) | .66 | .51  [-.11, .22] | -.04  (.08) | -.44 | .66  [-.19, .12] |
| CIT:  Time _pre/post_ | -.12  (.08) | -1.46 | .15  [-.27, .04] | 0  (.11) | -.02 | .99  [-.21, .21] | -.02  (.08) | -.23 | .82  [-.17, .13] | -.13  (.07) | -1.77 | .08  [-.27, .01] |
| SW:  Time _pre/post_ | -.16  (.09) | -1.87 | .06  [-.33, .00] | **-.3**  **(.12)** | **-2.52** | **.01***  **[-.52, .07]** | -.04  (.08) | -.52 | .61  [-.20, .12] | .15  (.08) | 1.96 | .05  [.00, .30] |
| Age:  Time  _pre/post_ | .15  (.07) | 2.18 | .03  [.02, .28] | .18  (.09) | 2.02 | .05  [.01, .36] | .07  (.07) | .98 | .33  [-.06, .19] | .05  (.06) | .89 | .37  [-.06, .17] |
| IQ:  Time _pre/post_ | .02  (.07) | .27 | .79  [-.11, .15] | -.12  (.09) | -1.24 | .22  [-.28, .06] | .02  (.06) | .31 | .75  [-.10, .14] | -.06  (.06) | -1.04 | .31  [-.18, .05] |
| Gender:  Time _pre/post_ | -.16  (.07) | -2.36 | .02  [-.29, -.03] | .06  (.09) | .69 | .49  [-.11, .23] | -.06  (.06) | -1.02 | .31  [-.19, .06] | -.12  (.06) | -1.94 | .05  [-.23, -.00] |

*IP, Information Processing; AD, Anxious-Depression; CIT, Compulsive behavior and Intrusive Thought; SW, Social Withdrawal; SE, Standard Error. Uncorrected p values are shown in the table. The main text reports in addition p values FDR-corrected FDR-corrected over the number of dependent variables (N = 4), asterisks denote FDR-corrected significance *p<.05, ** p<.01, ***p<.001*

**Table S4.** Results from linear mixed models examining longitudinal changes in survey-related measures of self-regulation

|  | **Agreeableness** | | | **Emotional Control** | | | **Ethical Risk-Taking** | | | **Goal-directed /Mindfulness** | | |
| --- | --- | --- | --- | --- | --- | --- | --- | --- | --- | --- | --- | --- |
|  | **β**  **(SE)** | **t value** | **p value** | **β**  **(SE)** | **t value** | **p value** | **β**  **(SE)** | **t value** | **p value** | **β**  **(SE)** | **t value** | **p value** |
| Intercept | -.03 (.08) | -.35 | .73  [-.18, .12] | -.01  (.05) | -.18 | .86  [-.10, .09] | .03  (.08) | .42 | .66  [-.12, .18] | -.03  (.07) | -.47 | .64  [-.16, .09] |
| AD | **-.5**  **(.11)** | **-4.69** | **<.001*****  **[-.71, -.30]** | **-.43**  **(.07)** | **-6.39** | **<.001*****  **[-.56, -.30]** | .04  (.11) | .33 | .74  [-.17, .24] | **-.76**  **(.09)** | **-8.43** | **<.001*****  **[-.93, -.58]** |
| CIT | .08  (.10) | .85 | .40  [-.10, .27] | **-.24**  **(.06)** | **-3.97** | **<.001*****  **[-.36, -.13]** | **.29**  **(.10)** | **2.96** | **<.001*****  **[.10, .47]** | .01  (.08) | .13 | .9  [-.14, .17] |
| SW | .20  (.10) | 1.91 | .06  [.00, .39] | **-.41**  **(.06)** | **-6.3** | **<.001*****  **[-.53, -.29]** | .03  (.10) | .26 | .80  [-.17, .22] | .13  (.09) | 1.56 | .12  [-.03, .30] |
| Age | -.03  (.08) | -.37 | .71  [-.19, .13] | .02  (.05) | .36 | .72  [-.08, .12] | -.2  (.08) | -2.47 | .02  [-.36, -.05] | .08  (.07) | 1.18 | .24  [-.05, .21] |
| IQ | -.08  (.08) | -.94 | .35  [-.23, .08] | -.05  (.05) | -1.00 | .32  [-.15, .05] | .13  (.08) | 1.68 | .1  [-.02, .29] | -.13  (.07) | -1.98 | .05  [-.26, -.01] |
| Gender | .11  (.08) | 1.38 | .17  [-.04, .26] | **-.13**  **(.05)** | **-2.59** | **.01***  **[-.23, -.03]** | **-.23**  **(.08)** | **-2.91** | **<.001*****  **[-.39, -.08]** | -.02  (.07) | -.32 | .75  [-.15, .11] |
| Time _pre/post_ | -.02  (.06) | -.34 | .74  [-.13, .09] | .01  (.05) | .14 | .89  [-.08, .09] | -.02  (.06) | -.26 | .79  [-.13, .10] | -.01  (.04) | -.26 | .8  [-.09, .07] |
| AD:  Time _pre/post_ | -.10  (.08) | -1.27 | .21  [-.25, .05] | -.03  (.06) | -.49 | .63  [-.15, .09] | .06  (.08) | .77 | .44  [-.10, .22] | -.08  (.05) | -1.54 | .13  [-.19, .02] |
| CIT:  Time _pre/post_ | .03  (.07) | .41 | .69  [-.11, .17] | .03  (.06) | .53 | .59  [-.08, .14] | -.04  (.07) | -.5 | .62  [-.18, .11] | -.06  (.05) | -1.14 | .26  [-.15, .04] |
| SW:  Time _pre/post_ | .08  (.08) | 1.01 | .31  [-.07, .23] | -.06  (.06) | -.93 | .36  [-.17, .06] | .05  (.08) | .63 | .53  [-.10, .20] | -.03  (.05) | -.50 | .62  [-.13, .07] |
| Age:  Time | .10  (.06) | 1.61 | .11  [-.02, .21] | -.01  (.05) | -.16 | .87  [-.10, .08] | .11  (.06) | 1.77 | .08  [-.01, .23] | .04  (.04) | .92 | .36  [-.04, .12] |
| IQ:  Time _pre/post_ | .14  (.06) | 2.29 | .02  [.02, .25] | .03  (.05) | .7 | .49  [-.06, .12] | -.1  (.06) | -1.69 | .09  [-.22, .01] | .02  (.04) | .54 | .59  [-.06, .10] |
| Gender:  Time _pre/post_ | .07  (.06) | 1.21 | .23  [-.04, .19] | .03  (.05) | .56 | .56  [-.06, .12] | -.04  (.06) | -.59 | .56  [-.15, .08] | .06  (.04) | 1.37 | .18  [-.02, .13] |

|  | **Reward Sensitivity** | | | **Risk Perception** | | | **Sensation Seeking** | | | **Social Risk Taking** | | |
| --- | --- | --- | --- | --- | --- | --- | --- | --- | --- | --- | --- | --- |
|  | **β**  **(SE)** | **t value** | **p value** | **β**  **(SE)** | **t value** | **p value** | **β**  **(SE)** | **t value** | **p value** | **β**  **(SE)** | **t value** | **p value** |
| Intercept | .01 (.07) | .13 | .89  [-.13, .15] | -.07  (.08) | -.81 | .42  [-.23, .09] | .01  (.09) | .16 | .88  [-.15, .18] | .04  (.08) | .47 | .64  [-.11, .19] |
| AD | .05  (.10) | .53 | .60  [-.14, .24] | **-.34**  **(.12)** | **-2.95** | **<.001*****  **[-.56, -.12]** | .14  (.12) | 1.21 | .23  [-.08, .37] | -.15  (.11) | -1.39 | .17  **[-.35, .06]** |
| CIT | **.45**  **(.09)** | **4.94** | **<.001*****  **[.27, .62]** | .16  (.10) | 1.49 | .14  [-.04, .36] | **.32**  **(.11)** | **2.96** | **<.001*****  **[.11, .53]** | **.52**  **(.10)** | **5.38** | **<.001*****  **[.34, .71]** |
| SW | **-.61**  **(.10)** | **-6.38** | **<.001*****  **[-.79, -.43]** | .2  (.11) | 1.81 | .07  [-.01, .41] | **-.43**  **(.11)** | **-3.76** | **<.001*****  **[-.65, -.21]** | **-.41**  **(.10)** | **-4.01** | **<.001*****  **[-.61, -.22]** |
| Age | 0  (.08) | .01 | .1  [-.15, .15] | .03  (.09) | .34 | .74  [-.14, .20] | .01  (.09) | .1 | .92  [-.17, .18] | .04  (.08) | .43 | .67  [-.12, .19] |
| IQ | -.15  (.07) | -1.98 | .05  [-.29, -.01] | -.13  (.09) | -1.52 | .13  [-.29, .03] | .1  (.09) | 1.15 | .25  [-.07, .27] | -.03  (.08) | -.40 | .69  [-.19, .12] |
| Gender | -.13  (.07) | -1.78 | .08  [-.28, .01] | .12  (.09) | 1.34 | .18  [-.05, .28] | **-.25**  **(.09)** | **-2.77** | **.01***  **[-.42, -.08]** | .09  (.08) | 1.16 | .25  [-.06, .25] |
| Time _pre/post_ | .01  (.06) | .10 | .92  [-.10, .11] | -.01  (.06) | -.2 | .85  [-.14, .11] | 0  (.05) | .05 | .96  [-.10, .11] | 0  (.06) | -.08 | .94  [-.12, .11] |
| AD:  Time _pre/post_ | -.09  (.08) | -1.13 | .26  [-.23, .06] | .06  (.09) | .74 | .46  [-.10, .23] | -.09  (.07) | -1.18 | .24  [-.23, .06] | -.04  (.08) | -.49 | .62  [-.20, .12] |
| CIT:  Time _pre/post_ | .1  (.07) | 1.43 | .15  [-.03, .23] | -.06  (.08) | -.7 | .49  [-.21, .10] | .15  (.07) | 2.22 | .03  [.02, .28] | .14  (.08) | 1.89 | .06  [.00, .29] |
| SW:  Time _pre/post_ | -.04  (.07) | -.61 | .55  [-.18, .10] | .06  (.08) | .73 | .47  [-.10, .22] | -.07  (.07) | -.98 | .33  [-.21, .07] | -.05  (.08) | -.62 | .53  [-.21, .10] |
| Age:  Time | -.05  (.06) | -.94 | .35  [-.17, .06] | -.09  (.07) | -1.28 | .2  [-.21, .04] | -.05  (.06) | -.92 | .36  [-.16, .06] | .04  (.06) | .68 | .49  [-.08, .17] |
| IQ:  Time _pre/post_ | -.06  (.06) | -1.02 | .31  [-.17, .05] | -.12  (.07) | -1.85 | .07  [-.25, .00] | -.04  (.06) | -.64 | .52  [-.14, .07] | -.09  (.06) | -1.35 | .18  [-.21, .04] |
| Gender:  Time _pre/post_ | .03  (.06) | .49 | .62  [-.08, .14] | -.00  (.07) | -.08 | .94  [-.13, .12] | .03  (.06) | .47 | .64  [-.08, .13] | -.01  (.06) | -.14 | .89  [-.13, .11] |

*AD, Anxious-Depression; CIT, Compulsive behavior and Intrusive Thought; SW, Social Withdrawal; SE, Standard Error. Uncorrected p values are shown in the table. The main text reports p values FDR-corrected over the number of dependent variables (N = 8), asterisks denote FDR-corrected significance *p<.05, ** p<.01, ***p<.001*

**Table S5.** Results from linear mixed models examining the trajectories of wellbeing related to the pandemic onset

|  | **Perceived stress** | | | **Loneliness** | | | **Social support** | | |
| --- | --- | --- | --- | --- | --- | --- | --- | --- | --- |
|  | **β**  **(SE)** | **t value** | **p value**  **[95% CI]** | **β**  **(SE)** | **t value** | **p value**  **[95% CI]** | **β**  **(SE)** | **t value** | **p value**  **[95% CI]** |
| Intercept | -.01  (.05) | -.12 | .90  [-.10, .09] | .01  (.07) | .08 | .94  [-.13, .14] | -.03  (.08) | -.41 | .68  [-.18, .11] |
| Mindset stress | .06  (.05) | 1.08 | .28  [-.04, .16] | -.14  (.08) | -1.83 | .07  [-.28, .01] | .12  (.08) | 1.47 | .14  [-.04, .28] |
| Mindset pandemic | -.03  (.05) | -.61 | .54  [-.14, .07] | .06  (.08) | .77 | .44  [-.09, .20] | .1  (.08) | 1.28 | .20  [-.05, .26] |
| AD | **.52**  **(.07)** | **7.37** | **<.001*****  **[.38, .65]** | **.30**  **(.10)** | **3.03** | **<.001*****  **[.11, .48]** | **-.52**  **(.11)** | **-4.89** | **<.001*****  **[-.72, -.32]** |
| CIT | **.26**  **(.06)** | **4.04** | **<.001*****  **[.14, .38]** | .18  (.09) | 2.05 | .04  [.01, .35] | -.02  (.10) | -.23 | .82  [-.20, .16] |
| SW | **.2**  **(.07)** | **2.99** | **<.001*****  **[.07, .33]** | .18  (.09) | 1.96 | .05  [.01, .36] | -.16  (.10) | -1.56 | .12  [-.35, .03] |
| Age | .06  (.05) | 1.07 | .29  [-.04, .16] | .05  (.07) | .71 | .48  [-.09, .19] | .01  (.08) | .18 | .86  [-.14, .17] |
| Gender | .01  (.05) | .10 | .92  [-.09, .10] | -.12  (.07) | -1.69 | .09  [-.26, .02] | .08  (.08) | 1.04 | .30  [-.07, .23] |
| Time _pre/post_ | -.02  (.07) | -.25 | .80  [-.15, .12] | -.01  (.09) | -.12 | .9  [-.18, .15] | -.01  (.04) | -.19 | .85  [-.08, .07] |
| Mindset stress: Time _pre/post_ | .08  (.08) | 1.11 | .27  [-.06, .23] | .11  (.09) | 1.22 | .22  [-.06, .29] | -.02  (.04) | -.53 | .60  [-.10, .06] |
| Mindset pandemic: Time _pre/post_ | .07  (.07) | .93 | .36  [-.07, .21] | .21  (.09) | 2.25 | .03  [.03, .38] | -.06  (.04) | -1.36 | .18  [-.14, .02] |
| AD:  Time _pre/post_ | -.04  (.1) | -.45 | .65  [-.23, .14] | -.19  (.12) | -1.56 | .12  [-.42, .04] | -.07  (.05) | -1.33 | .19  [-.18, .03] |
| CIT:  Time _pre/post_ | .02  (.09) | .2 | .84  [-.15, .18] | .07  (.11) | .61 | .54  [-.14, .27] | 0  (.05) | .01 | .99  [-.09, .09] |
| SW:  Time _pre/post_ | .03  (.09) | .32 | .75  [-.15, .20] | .03  (.11) | .29 | .77  [-.18, .25] | .05  (.05) | 1.05 | .30  [-.04, .15] |
| Age:  Time _pre/post_ | .11  (.07) | 1.45 | .15  [-.03, .25] | .09  (.09) | .99 | .33  [-.08, .26] | .03  (.04) | .70 | .48  [-.05, .11] |
| Gender:  Time _pre/post_ | -.04  (.07) | -.54 | .59  [-.17, .10] | -.08  (.09) | -.91 | .36  [-.25, .09] | .03  (.04) | .80 | .43  [-.04, .11] |

*AD, Anxious-Depression; CIT, Compulsive behavior and Intrusive Thought; SW, Social Withdrawal; SE, Standard Error. Uncorrected p values are shown in the table. The main text reports p values FDR-corrected over the number of dependent variables (N = 3), asterisks denote FDR-corrected significance *p<.05, ** p<.01, ***p<.001*

**Table S6.** Results from linear models examining the subjective impact of the pandemic onset

|  | **COVID-19 worries** | | | **Changes relationship** | | | **Economic concern** | | | **General anxiety** | | |
| --- | --- | --- | --- | --- | --- | --- | --- | --- | --- | --- | --- | --- |
|  | **β**  **(SE)** | **t value** | **p value**  **[95% CI]** | **β**  **(SE)** | **t value** | **p value**  **[95% CI]** | **β**  **(SE)** | **t value** | **p value**  **[95% CI]** | **β**  **(SE)** | **t value** | **p value**  **[95% CI]** |
| Intercept | -.01  (.07) | -.19 | .85  [-.14, .12] | 00  (.06) | -.06 | .95  [-.13, .12] | -.02  (.06) | -.28 | .78  [-.14, .11] | .03  (.06) | .48 | .63  [-.09, .15] |
| Mindset stress | -.13  (.07) | -1.82 | .07  [-.27, .01] | **.20**  **(.07)** | **3.07** | **<.001*****  **[.07, .33]** | .08  (.07) | 1.18 | .24  [-.05, .22] | -.07  (.06) | -1.1 | .27  [-.20, .06] |
| Mindset pandemic | **.21**  **(.07)** | **3.01** | **.001*****  **[.07, .35]** | .03  (.07) | .44 | .66  [-.10, .16] | -.06  (.07) | -.87 | .39  [-.19, .08] | .07  (.06) | 1.1 | .27  [-.06, .20] |
| AD | -.21  (.09) | -2.28 | .02  [-.39, -.03] | **-.29**  **(.08)** | **-3.42** | **<.001*****  **[-.46, -.12]** | .16  (.09) | 1.81 | .07  [-.01, .34] | .09  (.08) | 1.12 | .26  [-.07, .26] |
| CIT | **.45**  **(.08)** | **5.52** | **<.001*****  **[.29, .62]** | -.09  (.08) | -1.17 | .25  [-.24, .06] | **.19**  **(.08)** | **2.42** | **.02***  **[.04, .35]** | **.28**  **(.07)** | **3.72** | **<.001*****  **[.13, .42]** |
| SW | .06  (.09) | .75 | .46  [-.11, .24] | -.05  (.08) | -.62 | .54  [-.21, .11] | -.03  (.08) | -.35 | .73  [-.20, .14] | **.28**  **(.08)** | **3.61** | **<.001*****  **[.13, .44]** |
| Age | .00  (.07) | .04 | .97  [-.13, .14] | .09  (.06) | 1.35 | .18  [-.04, .21] | -.06  (.07) | -.87 | .39  [-.19, .07] | .08  (.06) | 1.27 | .21  [-.04, .20] |
| Gender | .00  (.07) | .00 | 1  [-.13, .13] | .16  (.06) | 2.6 | .01  [.04, .29] | .00  (.07) | -.07 | .94  [-.13, .12] | .06  (.06) | .9 | .37  [-.07, .18] |

|  | **Media usage** | | | **Negative mood** | | | **Physical exercise** | | | **Sleep hours** | | |
| --- | --- | --- | --- | --- | --- | --- | --- | --- | --- | --- | --- | --- |
|  | **β**  **(SE)** | **t value** | **p value**  **[95% CI]** | **β**  **(SE)** | **t value** | **p value**  **[95% CI]** | **β**  **(SE)** | **t value** | **p value**  **[95% CI]** | **β**  **(SE)** | **t value** | **p value**  **[95% CI]** |
| Intercept | .01  (.06) | .11 | .91  [-.11, .13] | .01  (.03) | .22 | .82  [.06, .08] | .03  (.07) | .49 | .62  [-.10, .16] | -.01  (.07) | -.09 | .93  [-.14, .13] |
| Mindset stress | .02  (.07) | .32 | .75  [-.11, .15] | -.08  (.04) | -2.23 | .03  [-.16, -.01] | .07  (.07) | 1.02 | .31  [-.07, .21] | -.07  (.07) | -.92 | .36  [-.21, .08] |
| Mindset pandemic | .13  (.06) | 2.07 | .04  [.01, .26] | -.04  (.04) | -1.11 | .27  [-.12, .03] | -.04  (.07) | -.62 | .54  [-.18, .10] | **-.30**  **(.07)** | **-4.17** | **<.001*****  **[-.44, -.16]** |
| AD | .08  (.08) | .93 | .35  [-.09, .25] | **.31**  **(.05)** | **6.49** | **<.001*****  **[.22, .41]** | -.19  (.09) | -2.06 | .04  [-.37, -.01] | .03  (.09) | .33 | .74  [-.15, .22] |
| CIT | **.20**  **(.08)** | **2.58** | **.01***  **[.05, .35]** | **.49**  **(.044)** | **11.11** | **<.001*****  **[.40, .57]** | **.18**  **(.08)** | **2.16** | **.03***  **[.02, .34]** | -.10  (.08) | -1.19 | .24  [-.27, .07] |
| SW | .06  (.08) | .76 | .45  [-.10, .22] | **.24**  **(.05)** | **5.17** | **<.001*****  **[.15, .33]** | -.13  (.09) | -1.47 | .14  [-.30, .04] | -.10  (.09) | -1.11 | .27  [-.28, .08] |
| Age | .10  (.06) | 1.62 | .11  [-.02, .23] | -.01  (.04) | -.21 | .83  [-.08, .06] | .09  (.07) | 1.36 | .17  [-.04, .23] | .00  (.07) | .04 | .97  [-.14, .14] |
| Gender | -.06  (.06) | -.94 | .35  [-.18, .06] | .07  (.04) | 1.94 | .05  [.00, .14] | .03  (.07) | .44 | .66  [-.10, .16] | .00  (.07) | -.05 | .96  [-.14, .13] |

|  | **Sleep time** | | | **Stress life changes** | | |
| --- | --- | --- | --- | --- | --- | --- |
|  | **β**  **(SE)** | **t value** | **p value**  **[95% CI]** | **β**  **(SE)** | **t value** | **p value**  **[95% CI]** |
| Intercept | .03  (.07) | .4 | .69  [-.11, .16] | -.04  (.06) | -.73 | .47  [-.15, .07] |
| Mindset stress | -.05  (.07) | -.74 | .46  [-.20, .09] | .02  (.06) | .36 | .72  [-.10, .14] |
| Mindset pandemic | .04  (.07) | .61 | .55  [-.10, .19] | .05  (.06) | .87 | .39  [-.07, .17] |
| AD | .20  (.09) | 2.13 | .03  [.02, .39] | -.04  (.08) | -.57 | .57  [-.20, .11] |
| CIT | .14  (.09) | 1.68 | .09  [-.03, .31] | **.36**  **(.07)** | **5.04** | **<.001*****  **[.22, .50]** |
| SW | -.08  (.09) | -.89 | .37  [-.26, .10] | .08  (.07) | 1.05 | .3  [-.07, .23] |
| Age | .11  (.07) | 1.49 | .14  [-.03, .25] | .12  (.06) | 2.13 | .03  [.01, .24] |
| Gender | -.13  (.07) | -1.82 | .07  [-.27, .01] | -.07  (.06) | -1.25 | .21  [-.19, .04] |

*AD, Anxious-Depression; CIT, Compulsive behavior and Intrusive Thought; SW, Social Withdrawal; SE, Standard Error. Uncorrected p values are shown in the table. The main text reports p values FDR-corrected over the number of dependent variables (N = 10), asterisks denote FDR-corrected significance *p<.05, ** p<.01, ***p<.001*

**Table S7.** Prospective predictions. Ridge regression using pre-onset COVID-19 factor scores to predict individual psychiatric symptoms.

|  |  | Apathy | Schizotypy | Social Anxiety | Eating Disorder | Alcohol disorder | Anxiety | Depression | Impulsivity | OCD |
| --- | --- | --- | --- | --- | --- | --- | --- | --- | --- | --- |
| **Tasks Factor Scores** | R^2^ | -0.6  (0.01) | -0.05  (0.02) | -0.09  (0.01) | -0.05  (0.02) | -0.07  (0.02) | -0.08  (0.0) | -0.07  (0.01) | -0.09  (0.0) | -0.11  (0.0) |
|  | MAE | 0.84  (0.82) | 0.8  (0.77) | 0.86  (0.82) | 0.8  (0.77) | 0.87  (0.84) | 0.85  (0.82) | 0.9  (0.87) | 0.78  (0.76) | 0.85  (0.8) |
| **Survey Factor Scores** | R^2^ | 0.33  (0.4) | 0.35  (0.45) | 0.34  (0.44) | 0.12  (0.24) | -0.09  (0.07) | 0.54  (0.61) | 0.31  (0.42) | 0.48  (0.57) | 0.26  (0.38) |
|  | MAE | 0.65  (0.61) | 0.58  (0.53) | 0.65  (0.6) | 0.71  (0.67) | 0.88  (0.82) | 0.52  (0.49) | 0.69  (0.63) | 0.56  (0.51) | 0.7  (0.64) |

*Insample score is displayed in parenthesis*

**Table S8.** Prospective predictions. Ridge regression using pre-onset COVID-19 factor scores to predict transdiagnostic dimensions of psychiatry.

|  |  | AD | CIT | SW |
| --- | --- | --- | --- | --- |
| **Tasks Factor Scores** | R^2^ | -0.07  (0.01) | -0.07  (0.01) | -0.07  (0.01) |
|  | MAE | 0.83  (0.8) | 0.73  (0.7) | 0.84  (0.81) |
| **Survey Factor Scores** | R^2^ | 0.48  (0.55) | 0.39  (0.52) | 0.41  (0.52) |
|  | MAE | 0.56  (0.52) | 0.52  (0.46) | 0.58  (0.53) |

*Insample score is displayed in parenthesis; AD, Anxious-Depression; CIT, Compulsive behavior and Intrusive Thoughts.*

**Table S9.** Cross-sectional predictions. Ridge regression using post-onset COVID-19 factor scores to predict individual psychiatric symptoms.

|  |  | Apathy | Schizotypy | Social Anxiety | Eating Disorder | Alcohol disorder | Anxiety | Depression | Impulsivity | OCD |
| --- | --- | --- | --- | --- | --- | --- | --- | --- | --- | --- |
| **Tasks Factor Scores** | R^2^ | -0.06  (0.02) | -0.05  (0.01) | -0.05  (0.03) | -0.02  (0.06) | -0.04  (0.03) | -0.07  (0.01) | -0.06  (0.01) | -0.09  (0.0) | -0.11  (0.01) |
|  | MAE | 0.85  (0.82) | 0.79  (0.77) | 0.83  (0.79) | 0.79  (0.75) | 0.86  (0.83) | 0.85  (0.82) | 0.9  (0.87) | 0.8  (0.76) | 0.84  (0.8) |
| **Survey Factor Scores** | R^2^ | 0.42  (0.51) | 0.48  (0.55) | 0.41  (0.5) | 0.07  (0.23) | -0.13  (0.04) | 0.64  (0.7) | 0.45  (0.53) | 0.52  (0.61) | 0.3  (0.4) |
|  | MAE | 0.59  (0.55) | 0.51  (0.48) | 0.6  (0.56) | 0.74  (0.68) | 0.9  (0.83) | 0.45  (0.41) | 0.59  (0.55) | 0.52  (0.47) | 0.66  (0.61) |

*Insample score in parenthesis*

**Table S10.** Cross-sectional predictions. Ridge regression using post-onset COVID-19 factor scores to predict to predict transdiagnostic dimensions of psychiatry

|  |  | AD | CIT | SW |
| --- | --- | --- | --- | --- |
| **Tasks Factor Scores** | R^2^ | -0.07  (0.01) | -0.04  (0.02) | -0.07  (0.04) |
|  | MAE | 0.83  (0.8) | 0.71  (0.69) | 0.83  (0.79) |
| **Survey Factor Scores** | R^2^ | 0.6  (0.66) | 0.49  (0.57) | 0.52  (0.6) |
|  | MAE | 0.43  (0.46) | 0.45  (0.49) | 0.49  (0.54) |

*Insample score is displayed in parenthesis; AD, Anxious-Depression; CIT, Compulsive behavior and Intrusive Thoughts.*

**Table S11.** List of cognitive tasks for self-regulation and derived variables

| **Task** | **Derived variables used for Exploratory Factor Analysis** |
| --- | --- |
| Adaptive N-Back | - DDM Parameters^1^ - Drift Rate as a function of load - Average load |
| Angling Risk Task | Two Conditions (Keep, Release):   - Adjusted Clicks - Coefficient of Variation (release condition) - Score |
| Attention Network Task | - DDM Parameters^1^​ - Alerting Effect - Orienting Effect - Conflict Effect |
| Bickel Titrator | - Discount Rate for three payout magnitudes |
| Choice Reaction Task | - DDM Parameters^1^​ |
| Cognitive Reflection Task | - Correct Proportion - Intuitive Proportion |
| Columbia Card Task Cold/Hot | - Average # of cards chosen - Gain Sensitivity - Loss Sensitivity​ - # Loss Cards Sensitivity - Level of Information Use |
| Dietary Decision Task | - Health Sensitivity - Taste Sensitivity |
| Digit Span | - Forward Span - Reverse Span |
| Directed Forgetting | - DDM Parameters ^1​^ - Proactive Interference |
| Discount Titrator | - Percent Patient |
| Dot Pattern Expectancy | - DDM Parameters ^1​^ - AY-BY - BX-BY - D-prime - Bias |
| Go-NoGo | - D-prime - Bias |
| Hierarchical Learning Task | - Total Score |
| Holt & Laury | - Percent Patient - Beta (inverse softmax temperature)​ - Risk Aversion (value function curvature) - # Safe Choices |
| Information Sampling Task | Two conditions (Decreasing Win, Fixed Win):   - Probability Correct at choice - Motivation |
| Keep Track Task | - Score |
| Kirby | - Discount Rate for three payout magnitudes - Percent Patient Choices - Percent Patient Choices for three payout magnitudes |
| Local-Global Task | - DDM Parameters ^1​^ - Switch Cost - Conflict Effect - Global Bias |
| Motor Selective Stop Signal | - DDM Parameters ^1​^ - SSRT - Reactive Control - Selective Proactive Control - Proactive Control |
| Probabilistic Selection Task | - Positive Learning Bias - Value Sensitivity |
| Psychological Refractory Period Task | - Slope of PRP function |
| Raven’s Progressive Matrices | - Score |
| Recent Probes | - DDM Parameters - Proactive Interference |
| Shape Matching Task | - DDM Parameters - Stimulus Interference |
| Shift Task | - Accuracy - Learning Rate - Learning to Learn   Model parameters:   - Beta (inverse softmax temperature) - Attentional Decay - RL learning rate |
| Simon Task | - DDM Parameters - Simon Effect |
| Simple Reaction Time | - Average Reaction Time |
| Spatial Span | - Forward Span - Reverse Span |
| Stimulus Selective Stop Signal | - DDM Parameters - SSRT - Reactive Control |
| Stop Signal | - DDM Parameters - SSRT (low stop signal probability condition) - SSRT (high stop signal probability condition)​ - Proactive SSRT speeding - Proactive Slowing |
| Stroop | - DDM Parameters - Stroop Effect |
| Cue/Task-Switch | - DDM Parameters - Stimulus Switch Cost - Task Switch Cost |
| Tower of London | - Average Move Time - # Extra Moves - # Optimal Solutions - Planning Time |
| Two-step Decision | - Model-Based Index - Model-Free Index - Perseverance |
| Writing Task | Sentiment Analysis:   - Positive Probability - Negative Probability |

1 ​DDM Parameters include drift rate, threshold and non-decision time

**Table S12.** List of surveys for self-regulation and derived variables

| **Self-Report Surveys** | **Derived variables used for Exploratory Factor Analysis** |
| --- | --- |
| BIS-BAS | - BAS Drive - BAS Fun-Seeking - BAS Reward-Responsiveness - BIS |
| Brief Self-Control Scale | - Self-Control |
| Dickman’s Impulsivity Inventory | - Functional |
| DOSPERT (EB/RP/RT) | - Ethical - Financial - Health/Safety - Recreational Social |
| Three-Factor Eating Questionnaire (R18) | - Cognitive Restraint - Emotional Eating - Uncontrolled Eating |
| Emotion Regulation Questionnaire | - Reappraisal - Suppression |
| Five Facet Mindfulness Questionnaire | - Acts with Awareness - Describe - Non judgment - Non reactive - Observe |
| Future Time Perspective | - Future Time Perspective |
| Grit Scale | - Grit |
| Impulsive-Venturesome Survey | - Venturesomeness - Impulsiveness |
| Mindful Attention Awareness Scale | - Mindfulness |
| Multidimensional Personality Questionnaire (Control subscale) | - Control​ |
| Selection Optimization Compensation | - Elective Selection - Loss-based Selection - Compensation - Optimization |
| Sensation Seeking Survey | - Boredom Susceptibility - Disinhibition - Experience Seeking - Thrill/Adventure Seeking |
| Short Self-Regulation Survey | - Control |
| Ten Item Personality Questionnaire | - Agreeableness - Conscientiousness - Emotional Stability - Extraversion - Openness |
| Theories of Willpower | - Endorse Limited Resource |
| Time Perspective Survey | - Past Positive - Past Negative - Present Hedonistic - Present Fatalistic - Future |
| UPPS+P | - Lack of Perseverance - Lack of Premeditation - Negative urgency - Positive urgency - Sensation seeking |

**Table S13.** List of surveys and items used to derive measures of health risk behavior

| **List of surveys and items used to derive measures of health risk behavior** |
| --- |
| - Weight |
| - Education level |
| - Relationship status |
| - Divorce count |
| - Years in relationship |
| - Number of relationships |
| - Number of children) |
| - Household income |
| - Retirement account |
| - Rent or Own a house |
| - Traffic tickets |
| - Traffic accidents |
| - Past or current problems with gambling |
| - Caffeine intake |
| - Legal troubles |
| - Alchol, smoking and drug questionnaire (Questions for alcohol, smoking and drugs were taken from the Alcohol Use Disorder Identification Test, Cannabis Use Disorder Identification test, and the Drug Abuse Screening test, respectively) |
| - Kessler psychological distress scale  (items referred to being nervous, hopeless, restless, depressed, feeling worthless, feeling that everything is an effort |

**References**

1. Eisenberg IW, Bissett PG, Enkavi AZ, Li J, MacKinnon DP, Marsch LA, Poldrack RA (2019): Uncovering the structure of self-regulation through data-driven ontology discovery. *Nat Commun* 10: 2319.

2. Zmigrod L, Eisenberg IW, Bissett PG, Robbins TW, Poldrack RA (2021): The cognitive and perceptual correlates of ideological attitudes: a data-driven approach. *Philos Trans R Soc B Biol Sci* 376: 20200424.

3. Rouault M, Seow T, Gillan CM, Fleming SM (2018): Psychiatric Symptom Dimensions Are Associated With Dissociable Shifts in Metacognition but Not Task Performance. *Biol Psychiatry*. https://doi.org/10.1016/j.biopsych.2017.12.017

4. Nikolaidis A, Paksarian D, Alexander L, Derosa J, Dunn J, Nielson DM, *et al.* (2021): The Coronavirus Health and Impact Survey (CRISIS) reveals reproducible correlates of pandemic-related mood states across the Atlantic [no. 1]. *Sci Rep* 11: 8139.

5. Yeo I-K, Johnson RA (2000): A New Family of Power Transformations to Improve Normality or Symmetry. *Biometrika* 87: 954–959.

6. Verbeke G, Molenberghs G (2000): *Linear Mixed Models for Longitudinal Data*. New York: Springer-Verlag. https://doi.org/10.1007/978-1-4419-0300-6

7. Wiecki TV, Sofer I, Frank MJ (2013): HDDM: Hierarchical Bayesian estimation of the Drift-Diffusion Model in Python. *Front Neuroinformatics* 7. https://doi.org/10.3389/fninf.2013.00014

8. Ratcliff R, Childers R (2015): Individual Differences and Fitting Methods for the Two-Choice Diffusion Model of Decision Making. *Decis Wash DC* 2015. Retrieved June 19, 2021, from https://www.ncbi.nlm.nih.gov/pmc/articles/PMC4517692/

9. Katahira K (2016): How hierarchical models improve point estimates of model parameters at the individual level. *J Math Psychol* 73: 37–58.

10. Enkavi AZ, Eisenberg IW, Bissett PG, Mazza GL, MacKinnon DP, Marsch LA, Poldrack RA (2019): Large-scale analysis of test–retest reliabilities of self-regulation measures. *Proc Natl Acad Sci* 116: 5472–5477.

11. Gillan CM, Kosinski M, Whelan R, Phelps EA, Daw ND (2016): Characterizing a psychiatric symptom dimension related to deficits in goal-directed control. *eLife* 5: e11305.

12. Marin RS, Biedrzycki RC, Firinciogullari S (1991): Reliability and validity of the Apathy Evaluation Scale. *Psychiatry Res* 38: 143–162.

13. Zung WW (1965): A SELF-RATING DEPRESSION SCALE. *Arch Gen Psychiatry* 12: 63–70.

14. Foa EB, Huppert JD, Leiberg S, Langner R, Kichic R, Hajcak G, Salkovskis PM (2002): The obsessive-compulsive inventory: development and validation of a short version. *Psychol Assess* 14: 485–496.

15. Patton JH, Stanford MS, Barratt ES (1995): Factor structure of the barratt impulsiveness scale. *J Clin Psychol* 51: 768–774.

16. Garner DM, Olmsted MP, Bohr Y, Garfinkel PE (1982): The eating attitudes test: psychometric features and clinical correlates. *Psychol Med* 12: 871–878.

17. Bates D, Mächler M, Bolker B, Walker S (2015): Fitting Linear Mixed-Effects Models Using lme4 [no. 1]. *J Stat Softw* 67: 1–48.

18. Nieuwenhuis R, Grotenhuis M te, Pelzer B (2012): influence.ME: Tools for Detecting Influential Data in Mixed Effects Models. *R J* 4: 38–47.

19. Vickers AJ, Altman DG (2001): Analysing controlled trials with baseline and follow up measurements. *BMJ* 323: 1123–1124.

20. Lakens D, Scheel AM, Isager PM (2018): Equivalence Testing for Psychological Research: A Tutorial. *Adv Methods Pract Psychol Sci* 1: 259–269.

21. Walker E, Nowacki AS (2011): Understanding Equivalence and Noninferiority Testing. *J Gen Intern Med* 26: 192–196.

22. Field A (2013): *Discovering Statistics Using IBM SPSS Statistics*. SAGE.

23. Browne MW, Cudeck R (1992): Alternative Ways of Assessing Model Fit. *Sociol Methods Res* 21: 230–258.
